# Wound state monitoring by multiplexed, electrochemical, real-time, localized, inflammation-tracking nitric oxide sensor (MERLIN)

**DOI:** 10.1126/sciadv.adv2385

**Published:** 2025-05-28

**Authors:** Liyang Wang, Yingqiao Wang, Mabel Bartlett, Daniel San Roman, Gaurav Balakrishnan, Samuel Gershanok, Reem Khan, Clint Skillen, Shanae Butler, Mangesh Kulkarni, Stephen F. Badylak, Devora Cohen-Karni, Bryan Brown, Tzahi Cohen-Karni

**Affiliations:** ^1^ Department of Materials Science and Engineering, Carnegie Mellon University, Pittsburgh, PA 15213, USA.; ^2^ Department of Biomedical Engineering, Carnegie Mellon University, Pittsburgh, PA 15213, USA.; ^3^ Department of Bioengineering, University of Pittsburgh, Pittsburgh, PA 15260, USA.; ^4^ McGowan Institute for Regenerative Medicine, University of Pittsburgh, Pittsburgh, PA 15219, USA.; ^5^ College of Osteopathic Medicine, Lake Erie College of Osteopathic Medicine, Greensburg, PA 15601, USA.

## Abstract

Nitric oxide (NO) released endogenously by induced NO synthase (iNOS) in macrophages is a key regulatory biomarker for wound inflammation. Detecting NO directly on the wound bed is challenging due to its short half-life time (6 to 50 seconds), low physiological concentration (nanomolar to micromolar), and interferences in the complex wound environment. Here, we present a compliant, multiplexed, electrochemical, real-time, localized, inflammation-tracking NO sensor (MERLIN) array for in vivo spatiotemporal measurement of NO, with high sensitivity (883 ± 283 nanoamperes per micromolar per square centimeter); selectivity against nitrites (~27,900-fold), ascorbic acid (~3800-fold), and uric acid (~6900-fold); and low limit of detection (~8.00 nM). MERLIN spatiotemporally tracked NO on rat skin wounds for 7 days, and results indicated that NO peaks on day 3, in line with previously reported iNOS activity. MERLIN allows spatial mapping of the NO gradient across the wound bed, which can be used to provide diagnostic information to assist wound care.

## Teaser

MERLIN allows real-time spatial mapping of the NO across the wound bed, which can be used to provide diagnostic information.

## INTRODUCTION

Nitric oxide (NO), a versatile and ubiquitous bioactive molecule, participates in multiple physiological processes, e.g., neurotransmission (*1*), neurovascular coupling (*2*), angiogenesis (*3*), and inflammation and immune response (*4*). As a small and uncharged free radical gaseous biomolecule, NO is able to freely diffuse across cell membranes. NO’s extra unpaired electron allows for highly reactive regulatory functions (*5*). NO modulates a variety of functions, e.g., activation of soluble guanylate cyclase in vasodilation (*6*), vesicle exocytosis and neurotransmitter release in neurotransmission (*1*, *7*), and phosphorylation of extracellular signal–regulated kinase in angiogenesis (*8*). In wound healing, proinflammatory cytokine release activates inducible NO synthase (iNOS) and increases endogenous production of NO (*9*), leading to activation of macrophages through *S*-nitrosylation (*10*, *11*), and direct DNA damage of pathogens via reactive nitrogen species such as peroxynitrite (*9*, *12*). Thus, NO serves as a biomarker for regulating various physiological processes, including monitoring the progression of the immune response during the inflammation stage of wound healing (*13*–*16*).

Wound healing is a complicated physiological process consists of four orchestrated and overlapping stages: hemostasis, inflammation, proliferation, and remodeling (*17*–*19*). The concentration change of signaling molecules, such as cytokines and chemokines, regulates and indicates the transition and completion of wound-healing stages (*20*, *21*). State-of-the-art wound sensors mainly monitor skin electrical impedance (*22*–*24*), pH (*25*), and temperature measurement (*26*). NO has a concentration-dependent characteristic that peaks in concentration during inflammation as part of the immune response for antimicrobial effects and decreases when transitioning to the proliferation stage (*13*). During inflammation, iNOS within the infiltrating neutrophils and macrophages at the wound bed catalyze oxidation of l-arginine amino acid (l-arg) to NO with a concentration ranging from nanomolar to micromolar (*27*–*29*). Measuring biochemical signaling molecules such as NO by a sensor will provide quantitative and potentially more comprehensive assessment of the dynamic wound-healing progress.

However, real-time in vivo detection of NO for wound-healing monitoring has yet to be achieved because of challenges including its low physiological concentration ranging from picomolar to micromolar (*30*), short half-life time (6 to 50 s) as a free radical in the biological scavenging environment (*31*, *32*), short diffusion distance (500 μm) (*31*, *33*), and the complex bio-environment consisting of ions, metabolic wastes, and interferences, leading to nonspecific adsorption and disturbing accurate measurement (*34*, *35*). To date, various in vivo NO detection approaches have been reported including optical resonance (*36*), fluorescence imaging (*37*), and electrochemical methods (*38*, *39*). Optical resonance measures NO as a function of refractive index changes and fluorescence imaging technique uses a fluorescent indicator for NO detection. Although these methods reported sub-micromolar NO concentration range, their applicability in real-time in vivo NO measurement is restricted by the rigid substrate and complex instrumentation. Electrochemical sensors offer a reliable alternative with advantages of superior temporal resolution, low limit of detection (LOD), and user-friendly setup (*38*, *39*). Nonetheless, state-of-the-art electrochemical NO sensor is limited to a single detection point and has yet to achieve in vivo wound-healing monitoring (table S1) (*40*, *41*).

To monitor the dynamics of wound healing, spatiotemporal assessment on wound bed informs NO sensor design requirements such as mechanical flexibility to allow conformal placement on wound bed for accurate detection of short-lived NO, compact device geometry, and high channel density to allow spatial monitoring, which can be readily achieved through microfabrication techniques. The design of a selective electrochemical sensor for endogenously produced NO requires the consideration of physical properties of NO, i.e., size, charge, and hydrophobicity. Redox active NO allows for direct oxidation on the working electrode (e.g., Pt), and the permeable membranes allow selective diffusion by effectively blocking out electrochemical interferences such as nitrites, ascorbic acids, and uric acids (*31*, *42*–*44*). When NO diffuses across selective layer and reaches the working electrode held at NO oxidation potential (e.g., 0.85 V for Pt), the oxidation of NO takes place via a two-step mechanism with an electrochemical reaction 1 that provides electronically readable signals, followed by a chemical reaction 2, described in the following reactions (*45*, *46*)

NO→NO++e− (1)

NO++OH−→HNO2 (2)

Among various electrochemical techniques, chronoamperometric (CA) detection of NO allows real-time capability with fast sampling rate and high sensitivity (*47*–*49*). The permselective modification renders specificity toward NO and CA transduces chemical concentration to electrical signals.

Here, we develop a thin and compliant, multiplexed, electrochemical, real-time, localized, inflammation-tracking NO sensor (MERLIN) array for in vivo NO spatiotemporal measurement, with high sensitivity and selectivity. We established a robust device fabrication protocol, along with comprehensive and highly reproducible in vitro characterization, confirming consistent device performance (i.e., sensitivity, selectivity, and LOD) for wound-healing monitoring. In addition, we used a rodent wound model to demonstrate temporal and spatial NO concentration mapping over the course of wound healing. The recorded NO concentrations matched previously reported literature, which informs inflammation trend of wound healing (*50*, *51*). By using the concentration profile of NO in normal versus chronic wound healing (*14*–*16*), MERLIN will enable real-time, reliable, and quantitative monitoring of wound state and alleviate subjectivity and dependence of visual expert wound assessment (*52*, *53*), thus facilitating chronic wound diagnosis, leading to improved wound care guidance and treatment outcomes.

## RESULTS AND DISCUSSION

### Compliant MERLIN array design

**Fig. 1.**
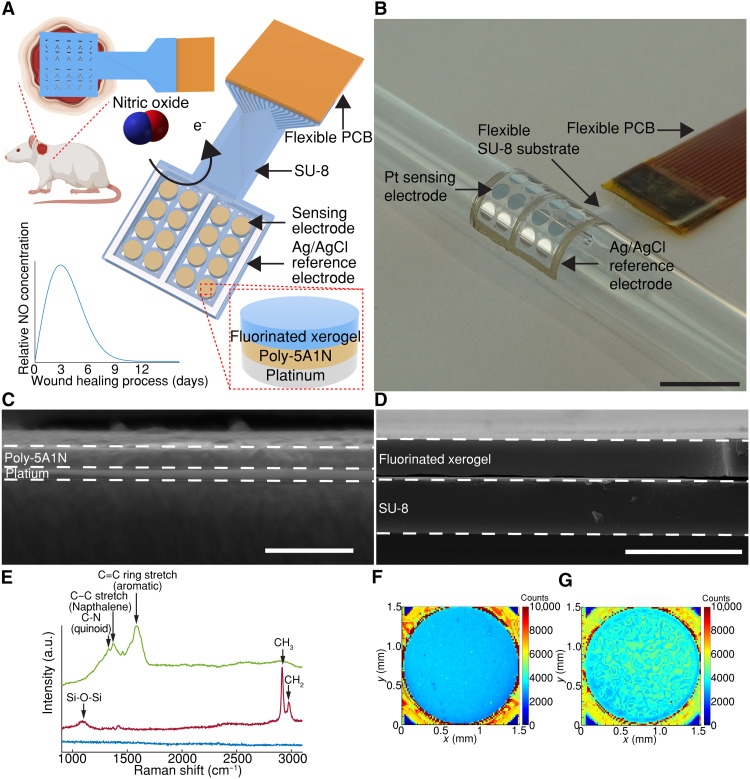
MERLIN for wound state monitoring. (**A**) Schematic of MERLIN array. e^−^, electron. (**B**) Photograph of conformable MERLIN array on a 7-mm–radius surface. Scale bar, 1 cm. (**C**) Representative cross-sectional electron microscopy image of poly-5A1N on Pt. Scale bar, 500 nm. (**D**) Representative cross-sectional electron microscopy of image of fluorinated xerogel on SU-8 by spray coating. Scale bar, 10 μm. (**E**) Representative Raman spectra of modified electrode surface. Blue, Pt; green, 5A1N; red, fluorinated xerogel; a.u. represents arbitrary units. (**F**) Representative Raman spectroscopy mapping of 5A1N-Pt electrode plotted at peak intensity of C=C. (**G**) Representative Raman spectroscopy mapping of fluorinated xerogel-5A1N-Pt electrode plotted at peak intensity of methyl group.

In vivo NO sensing requires a flexible array that conforms to the wound topography with measurement nodes within NO diffusion distance without impeding wound-healing progress (*31*, *33*, *54*). MERLIN array geometry allows for spatial mapping within a 2-cm–diameter rat skin wound model (Fig. 1A). The scalable, high–spatial resolution electrode array (4 by 4 array, electrode diameter of 1.5 mm with center-to-center distance of 2 mm) was fabricated following standard microfabrication techniques. Briefly, MERLIN arrays were patterned on a thin polymeric SU-8 substrate using photolithography, followed by metallization and passivation steps (for fabrication details, see the Materials and Methods, figs. S1 and S2). Last, to provide a stable electrochemical measurement in the complex wound environment, an on-chip silver/silver chloride (Ag/AgCl) reference electrode was screen printed on the fabricated arrays. The resulting ~10-μm–thick polymeric substrate and 100-nm thin film metal electrodes are mechanically flexible and can conform to as low as a 7-mm-radius surface (Fig. 1B), ensuring stable contact with the wound bed (fig. S3).

### Permselective layer for highly sensitive and selective NO measurement

High sensitivity and selectivity toward NO were achieved by permselective layers on the working electrode surface, allowing selective diffusion of NO toward the electrode by effectively blocking out electrochemical interferences such as nitrite, ascorbic acid, and uric acid (*31*, *42*–*44*). Permselective layers were selected based on size, charge, and hydrophobicity exclusion based on NO’s physical characteristics of small size, neutral charge, and lipophilicity (*31*, *42*, *43*, *45*, *55*). Inspired by previously published work, we adapted, optimized, and extensively characterized a bilayer structure of selective materials consisting of electrochemically polymerized 5-amino-1-naphthol (poly-5A1N) (fig. S4A) and spray-coated fluorinated xerogel reported in previous literature (*42*, *43*, *55*). The optimization of the deposition parameters resulted in selection of five cycles of cyclic voltammetry (CV) for fast 5A1N deposition (due to self-terminating characteristics with diminishing improvement in selectivity) and 10 s of spray coating to obtain a homogenous layer (fig. S5).

Electrochemically deposited 5A1N and spray-coated fluorinated xerogel exhibited a consistent thickness of 110 ± 3 nm and 2.04 ±0.17 μm, respectively (Fig. 1, C and D, and fig. S4B; for details, see Materials and Methods). The presence of poly-5A1N and fluorinated xerogel was confirmed via Raman spectroscopy (Fig. 1E). The presence of 5A1N’s polyaniline-like structures C–N, C–C, and C=C ring stretch (quinoid rings, naphthalene, and aromatic rings) with peaks at ~1330, 1370, and 1590 cm^−1^, respectively (*56*–*58*), supported its electrochemical polymerization. Cross-linked fluorinated xerogel polymeric coating of the electrode exhibited peaks at ~1060, 2840, and 2941 cm^−1^ corresponding to Si-O-Si network, methyl, and methylene groups, respectively (*59*, *60*). Both electrochemically deposited 5A1N and spray-coated fluorinated xerogel layers were uniform as can be seen in the Raman spectroscopy mapping (Fig. 1, F and G).

The NO oxidation peak was determined to be 0.82 ± 0.03 V (*n* = 3) by two-electrode system (fig. S6; for details, see the Materials and Methods), which is in good agreement with reported values (*43*, *61*). To thoroughly drive the NO oxidation reaction under CA and minimize the reference electrode chemical potential drift, an overpotential above the NO oxidation potential is applied. Thus, the MERLIN is operated at 0.85 V for CA measurement.

To understand the electrochemical properties of the modified electrodes, electrochemical impedance spectroscopy (EIS) was performed. EIS monitors electrode-electrolyte interface properties by applying a small amplitude of alternating potential over a wide range of frequencies and allows the investigation of physical processes at different time scales, e.g., from 500,000 Hz to 1 Hz. The collected EIS data were modeled with equivalent circuit elements that represent electrode-electrolyte interface, including solution resistance (*R*_s_), bulk capacitance (*C*_bulk_), double-layer capacitance (*C*_DL_), and electrode resistance (*R*_electrode_) (fig. S7) (*62*, *63*). An increase in *R*_s_ from 797 ± 240 ohms to 5.86 × 10^5^ ± 1.88 × 10^5^ ohms for Pt-(poly-5A1N) and Pt-(poly-5A1N)–fluorinated xerogel structure, respectively, was observed with deposition of fluorinated xerogel layer that is attributed to decrease in electrode surface accessibility (*n* ≥ 8; tables S2 and S3) (*64*). Such an increase in solution resistance was also reported in Poly(3,4-ethylenedioxythiophene)-poly(styrenesulfonate) (PEDOT:PSS)-coated nanowire-templated three-dimensional fuzzy graphene (*63*). A decrease of *C*_DL_ from 2.68 × 10^−6^ ± 1.41 × 10^−7^ to 7.32 × 10^−8^ ± 4.32 × 10^−8^ S-s^α^ for Pt and Pt-(poly-5A1N)–fluorinated xerogel electrodes, respectively, was attributed to the decrease in electrical conductivity of electrode and increased surface distance from Pt due to sequential addition of selective dielectric layers (table S2) (*65*). The decrease in capacitance is beneficial for amperometric sensors through decrease in the non-Faradaic baseline current and increases the signal-to-noise ratio (*66*, *67*).

To ensure the accuracy and stability of on-chip screen-printed Ag/AgCl electrodes, they were compared against commercial Ag/AgCl electrode as the working and reference electrode, respectively, by measuring CV in a three-electrode setup in the presence of 1 mM ${\left[\text{Fe}{\left(\text{CN}\right)}_{6}\right]}^{3-}$ solution. The CV half-wave potential difference between commercial Ag/AgCl and ink screen-printed Ag/AgCl on-chip electrode is 9.95 ± 0.28 mV (*n* = 3), which is ~1% difference compared to the amperometric operation at 0.85 V (fig. S8A). The stability of screen-printed Ag/AgCl electrodes shown by a drift of −0.273 ± 0.114 mV/hour (*n* = 3) obtained from a continuous 12-hour open-circuit potentiometry test (fig. S8, B and C) confirms steady electrochemical potential of the on-chip reference electrodes in our <12-hour planned experimental operation both in vitro and in vivo.

### Sensor performance: Sensitivity, selectivity, LOD, and stability

**Fig. 2.**
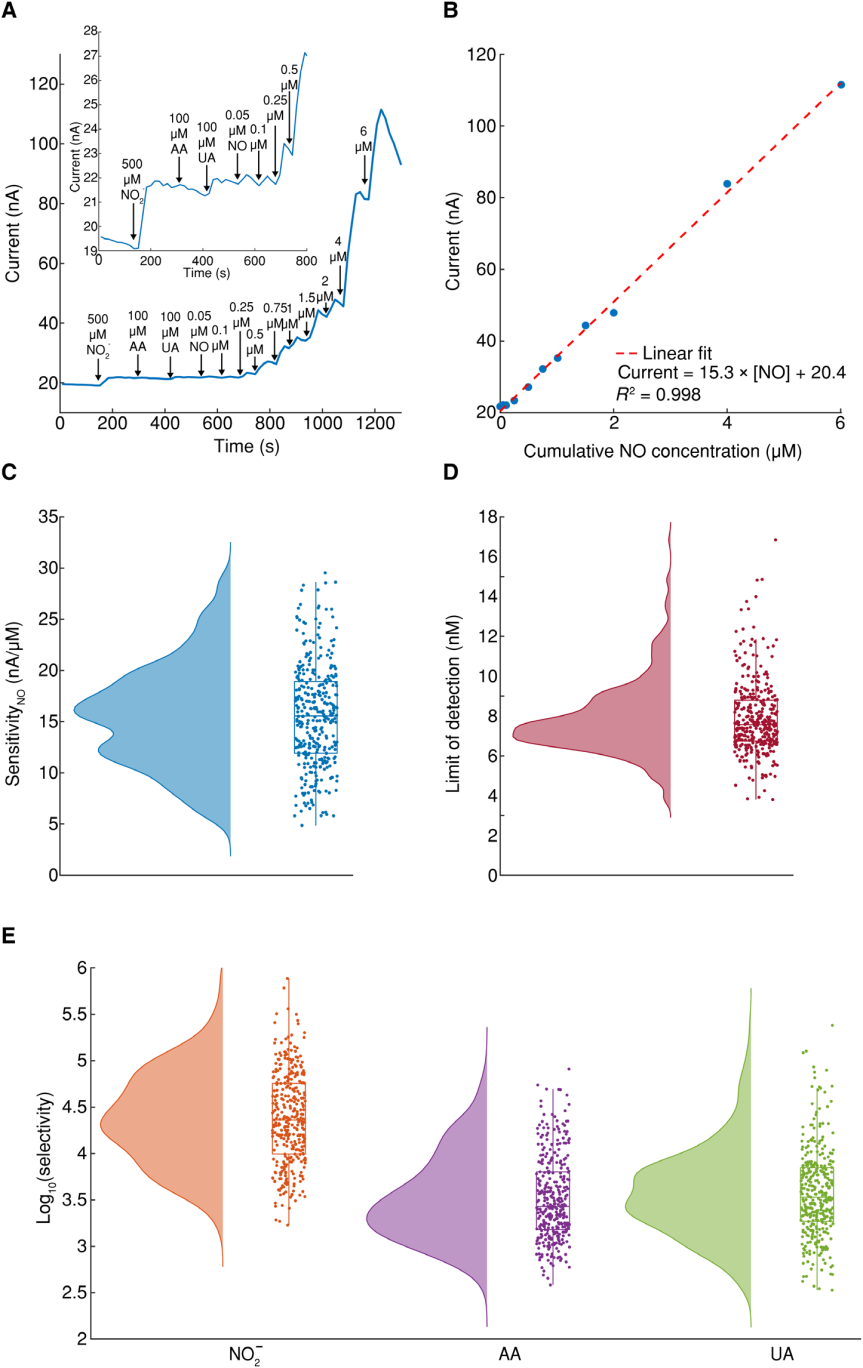
MERLIN in vitro calibration and sensing performance. (**A**) Representative single electrode current versus time with NO solution concentration changes. Typical interferants were added, i.e., nitrite (${\text{NO}}_{2}^{-}$), ascorbic acid (AA), and uric acid (UA). (**B**) Representative calibration curve of current versus NO concentration. (**C**) Sensitivity summary of MERLIN toward NO (*n* = 343). (**D**) LOD summary of MERLIN (*n* = 343). (**E**) Selectivity summary of MERLIN against common interferences such as nitrite (${\text{NO}}_{2}^{-}$), ascorbic acid (AA), and uric acid (UA) (*n* = 343); raincloud plots in (A) to (C) include half violin plots showing data distribution, boxplots, and raw data.

To evaluate the performance of MERLIN array, a standardized NO sensing calibration procedure was developed and deployed in deoxygenated phosphate-buffered saline (PBS) solution. Briefly, MERLIN was polarized, and baseline current was determined at 2 hours post-polarization. Subsequently, electrochemical interferences were added: nitrite (500 μM), ascorbic acid (100 μM), uric acid (100 μM), and NO solutions at physiologically relevant concentrations ranging from 50 nM to 6 μM (Fig. 2A and fig. S9A; for details, see the Materials and Methods). The solution was stirred to circumvent the diffusion of the reactants to the working electrode (fig. S9B; for details, see the Materials and Methods).

To quantify sensor performance, an electrical current versus time and NO concentration curves were obtained (Fig. 2, A and B). MERLIN demonstrated detection of NO within 2 s post–analyte solution addition, with a highly sensitive response (15.3 nA/μM) and a linear response up to 6 μM NO [coefficient of determination (*R*^2^) = 0.998], within the physiological NO concentration reported in literature (*30*). Selectivity against nitrite, ascorbic acid, and uric acid was calculated as the logarithmic ratio between sensitivity toward NO divided by sensitivity toward interference (for details, see the Materials and Methods). To allow reproducible and stable calibration, MERLIN sensor calibration was performed in PBS with eight-channel multiplexed measurement (fig. S10). We have further validated our calibration results using a simulated wound fluid (SWF) (*43*, *68*), imitating the protein content and ionic concentrations of wound exudate. No statistical significance was found in NO sensor performance in PBS as compared to SWF (fig. S11).

To evaluate sensor performance and reliable NO sensing, a large number (*n* = 343) of sensors were measured by using an eight-channel multiplexed system with reproducible and consistent results. MERLIN exhibits NO sensitivity of 15.6 ± 5.0 nA/μM (Fig. 2C, *n* = 343); LOD of 8.00 ± 2.37 nM (Fig. 2D, *n* = 343); and high selectivity against nitrite, ascorbic acid, uric acid at ~27,900 ± 2700, 3800 ± 500, and 6900 ± 800, respectively (Fig. 2E, *n* = 343). MERLIN demonstrates high sensitivity of 882 nA/μM per square centimeter, which is 26.8-fold higher than reported NO sensors (table S1). The high selectivity, sensitivity, comparable LOD, multichannel measurement with high spatial resolution, and extensive sensor calibration with reproducible results of MERLIN ensure accuracy of NO detection in vivo and further clinical applications.

### Tracking inflammation during wound healing

**Fig. 3.**
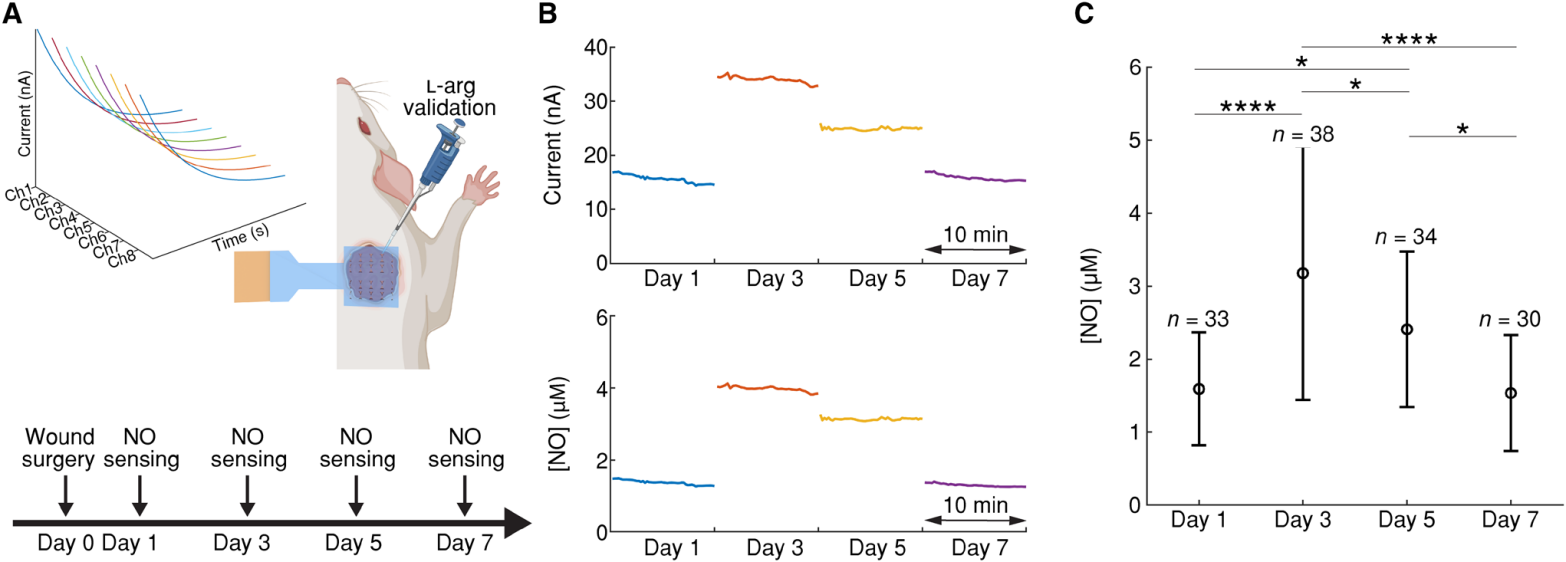
MERLIN in vivo rat skin wound inflammation monitoring. (**A**) Schematic illustration of NO sensing experiment in vivo, with sensing measurements on days 1, 3, 5, and 7 post-surgery. (**B**) Representative real-time monitoring of the current reading of MERLIN at different days post–wound surgery for rat 1. (**C**) MERLIN temporal NO measurement by each sensor with data shown as the means ± SDs. *P < 0.05 and ****P < 0.0001, based on one-way analysis of variance (ANOVA) and Tukey post hoc test.

To validate in vivo NO measurements, acute NO sensing measurements were performed on a rat skin wound model, on days 1, 3, 5, and 7 post-surgery (Fig. 3A). At each time point, a freshly calibrated and sterilized MERLIN array was placed directly on the wound to perform 1-hour measurement on anesthetized rats (fig. S12A). Real-time and continuous measurement of electrical current on the wound bed was obtained and converted to NO concentration (Fig. 3B and fig. S13; for details, see Materials and Methods) (*64*, *69*, *70*). To validate the functionality of MERLIN array, 25 μl of 10 mM l-arg, iNOS substrate (*71*, *72*), was added 30 min post-recording to simulate NO production and was followed by an increase in measured current (fig. S12C).

NO concentrations of 1.59 ± 0.77, 3.18 ± 1.74, 2.41 ± 1.07, and 1.54 ± 0.79 μM were detected on days 1, 3, 5, and 7 of wound healing, respectively, using MERLIN. The highest concentration of NO was observed on day 3, indicating a peak in the inflammation phase of wound healing (Fig. 3, B and C, and fig. S14). This observation is in line with previous literature on inflammation and NO concentration peak period as demonstrated by iNOS staining (*13*, *50*, *51*, *73*). NO is prominently produced by iNOS, activated by endogenous or exogenous danger signals such as damage-associated molecular patterns and pathogen-associated molecular patterns during inflammation stage, with peaks ranging from 0 to 4 days post–wound creation (*17*, *73*, *74*). Individual rat NO measurement also indicates an NO concentration peak on day 3 post-wounding, with three of the four rats (rats 1, 3, and 4) confirming this trend, indicating a repeatable and reliable inflammation tracking using MERLIN (Fig. 3C and fig. S14). Rat 2 was observed to have a delayed NO peak on day 5, which may be due to animal to animal variation in wound healing (*13*, *29*).

Given the importance of NO in the later stages of healing including collagen synthesis, cell proliferation, and wound contraction (*75*), future studies will aim to extend the monitoring period beyond 7 days to capture NO’s involvement in extracellular matrix remodeling and fibroblast-mediated tissue repair. MERLIN is a robust platform for continuous NO monitoring, making it feasible to investigate NO dynamics in chronic and delayed wound-healing models. By extending NO monitoring to later phases, NO’s regulatory functions throughout the wound-healing process can be elucidated, thus contributing to a more comprehensive understanding of NO as a biomarker and therapeutic target in wound care.

### MERLIN can spatially map the wound state

**Fig. 4.**
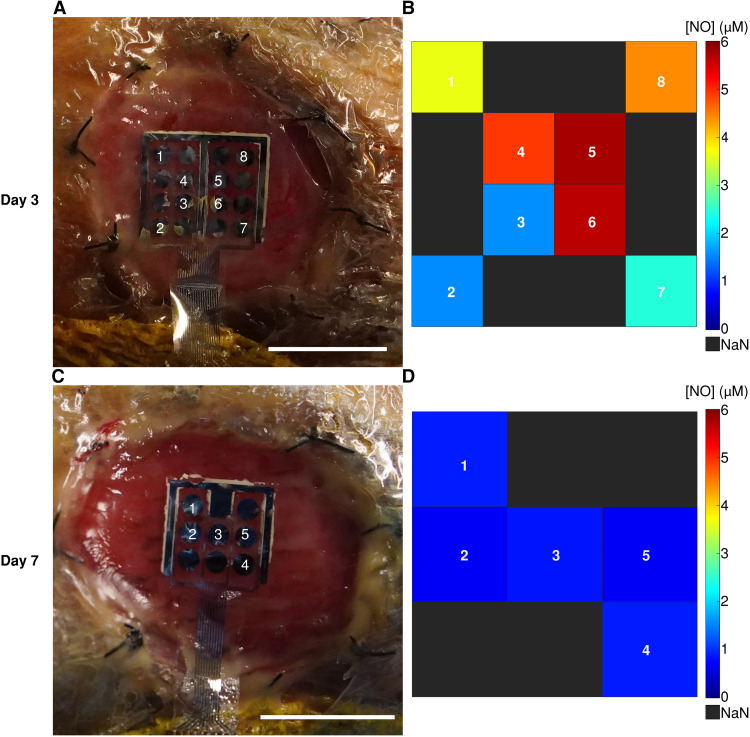
MERLIN spatial NO sensing in vivo. (**A**) Representative day 3 MERLIN NO measurement on rat skin wound in vivo. Scale bar, 1 cm. (**B**) NO concentration mapping readout. (**C**) Representative day 7 MERLIN NO measurement on rat skin wound in vivo. Scale bar, 1 cm. (**D**) NO concentration mapping readout.

A key advantage of MERLIN array is its ability to spatially map NO concentrations within the wound bed, which may provide information about the distribution of macrophages and iNOS activity (figs. S15 to S19). The NO concentration measurements obtained from MERLIN show varying NO concentration across the wound bed, which is expected because of the heterogeneous nature of the wound bed during the healing process as previously reported (*76*, *77*). The steep NO concentration gradient on day 3 (Fig. 4, A and B) suggested that more varied and up-regulated iNOS expression during the inflammatory phase of wound healing. The evenly distributed and lower NO concentration on day 7 (Fig. 4, C and D) may indicate the transition from the inflammatory to proliferation stage of wound healing with initiation of wound closure through down-regulation of iNOS by anti-inflammatory cytokines (*13*, *78*, *79*). MERLIN’s ability to spatiotemporally map NO may be beneficial for diagnosing chronic nonhealing wounds.

### MERLIN arrays are safe

**Fig. 5.**
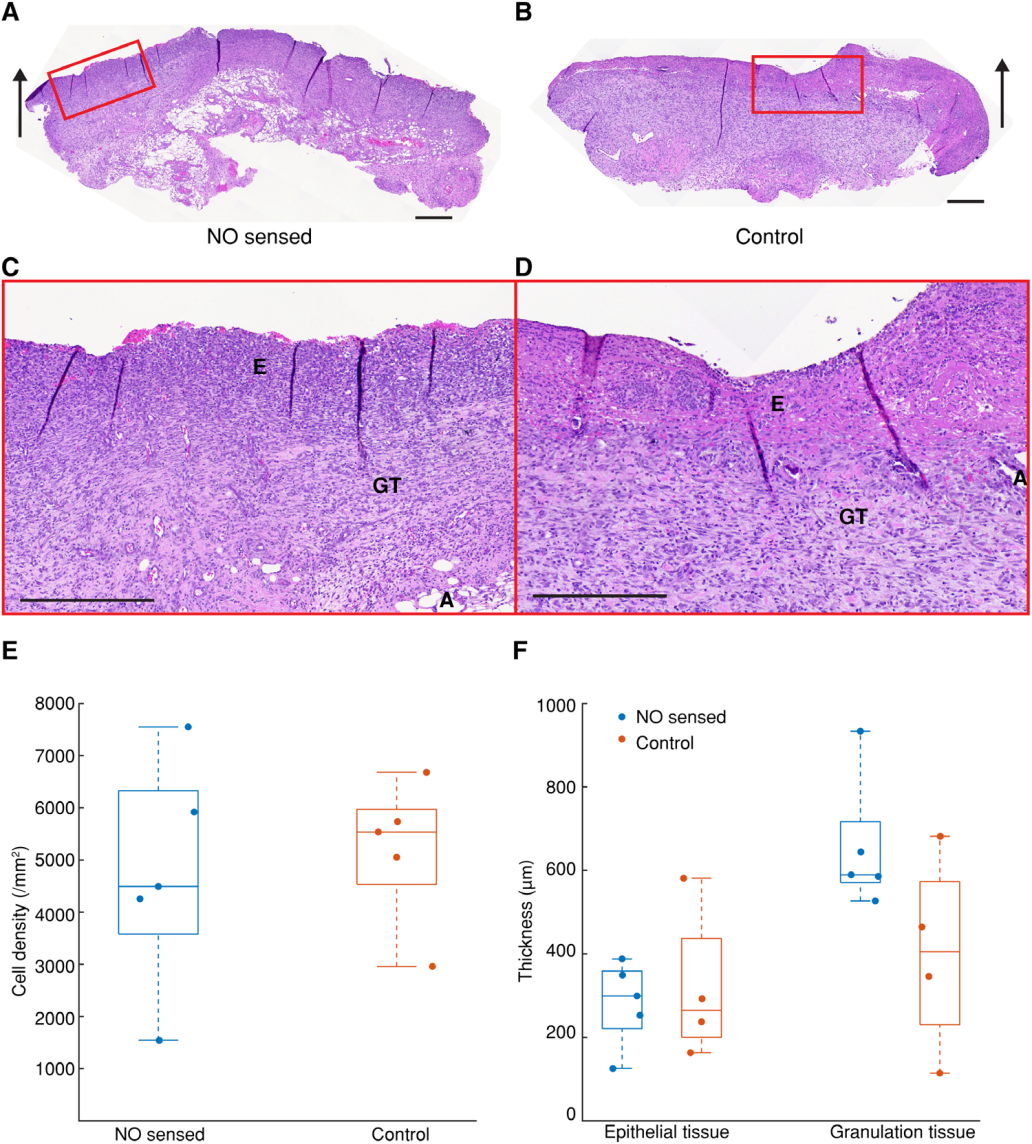
Tissue response to MERLIN. (**A**) Representative histological images of tissue harvested from rat skin wound with and (**B**) without MERLIN, at day 7 post-wounding. Both NO sensed wound and control wound show similar wound morphology. Black arrows show the direction of the wound surface. (**C**) Zoomed-in image of histological images of tissue with MERLIN NO sensing measurement. (**D**) Zoomed-in image of histological images of tissue without MERLIN NO sensing measurement. E, epithelium; GT, granulation tissue; A, adipose tissue (subcutaneous); scale bars, 400 μm. (**E**) Cell density of hematoxylin and eosin (H&E)–stained tissues does not show statistical significance based on one-way ANOVA and Tukey post hoc test, showing good biocompatibility (*n* = 5 rats). (**F**) Quantitative analysis of epithelial and granulation tissue thickness does not show statistical significance based on one-way ANOVA and Tukey post hoc test, showing good biocompatibility (*n* = 5 rats).

MERLIN arrays do not elicit adverse tissue response as demonstrated by hematoxylin and eosin (H&E) staining of histological tissue samples (day 7 post–wound creation). H&E-stained sections showed similar wound morphology of epithelium and granulation tissue (Fig. 5, A to D) in NO sensed and in control wounds, suggesting that the sensor placement and measurement did not affect the wound-healing process nor did it lead to additional inflammation. Cell density analysis showed a similar number of cell infiltrates observed with 5093 ± 1955 cells/mm^2^ and 5192 ± 1381 cells/mm^2^ in NO sensed and in control samples, respectively, confirming no adverse effects of the NO sensing measurements upon the inflammatory process or wound-healing outcome (Fig. 5E). Quantitative analysis of epithelial and granulation tissue thickness shows comparable thickness values in NO sensed and control samples, confirming no adverse effects of the NO sensing measurements upon the inflammatory process or wound-healing outcome (Fig. 5F). Additionally, the US Food and Drug Administration (FDA)–recommended biocompatibility endpoints were described in ISO 10993-1:2020, including cytotoxicity, sensitization, intracutaneous irritation, acute systemic toxicity, and pyrogenicity results demonstrate compliance with the biocompatibility standard requirements.

MERLIN is a compliant, multiplexed, real-time, highly sensitive, and selective NO sensor array with high sensitivity (883 nA/μM per square centimeter), high selectivity against electrochemical interference (27,900-fold against nitrite, 3800-fold against ascorbic acid, and 6900-fold against uric acid), spatiotemporal mapping capability, reproducible sensor performance, and no adverse effect on tissue or wound healing. MERLIN provides a practical diagnostic solution for quantitative wound monitoring to complement current practice of visual assessment of wound state. MERLIN can readily adapt its design and form factor to be integrable into a biohybrid electronic platform for feedback regulated wound healing with therapeutic guidance through monitoring of biochemical signals (*22*, *24*). MERLIN presents breakthrough in quantitative real-time measurement of the short-lived NO thus can enable the understanding (and diagnosis) of conditions related to vasodilation, neurovascular coupling, neurotransmission, angiogenesis, and immune response.

## MATERIALS AND METHODS

### MERLIN array microfabrication

MERLIN arrays were fabricated by standard clean room fabrication techniques. Briefly, Si/SiO_2_ (600 nm, Nova Electronic Materials, catalog no. CP02-11208-OX) was cleaned by sonication in acetone for 5 min, rinsed with isopropyl alcohol (IPA), and N_2_ blow dried. Wafers were treated in barrel etcher (IPC3000 Branson) with oxygen plasma at 850 mtorr, 100-W radio-frequency power for 1 min. The 5-μm-thick SU-8 (Kayaku Advanced Materials) was spin coated at 3000 rpm for 40 s before two-step pre-exposure bake at 65°C and 95°C respectively for 5 min. Ultraviolet (UV) exposure for photolithography was performed by MA6 (Karl Suss) at 5 mW/cm^2^ for 40 s followed by post-exposure bake at 65°C for 5 min and 95°C for 1 min. The micropatterned substrate was then developed in SU-8 developer (Kayaku Advanced Materials) for 5 min before hard baking at 190°C for 30 min. For the metal layer, a bilayer structure of 300-nm LOR3A (Kayaku Advanced Materials) and 1300-nm Shipley1813 (Kayaku Advanced Materials) was spin coated and baked at 190°C for 5 min and 115°C for 1 min, respectively. Photolithography was performed by mask aligner MA6 (Karl Suss) before development in 2.6% tetramethylammonium hydroxide aqueous solution (CD-26 Developer, Kayaku Advanced Materials) for 60 s. The pattern was deposited with a metal stack of 20-nm Cr and 80-nm Pt by DC sputtering at 50 W in a house-built sputtering system. Liftoff process was performed by using Remover PG (Kayaku Advanced Materials) at 60°C for 30 min. The patterned metal arrays were passivated with 5-μm SU-8 (Kayaku Advanced Materials), repeating the SU-8 bottom layer process.

### Ag/AgCl on-chip reference electrode screen printing

On-chip reference electrode was made by screen printing of Ag/AgCl ink (Creative Materials, catalog no. 126-49) by a micro paint brush (Flat 2, Nicpro), covered by 1-mil PET stencil (McMaster-Carr, catalog no. 8567 K12). Curing of Ag/AgCl ink was done at 150°C for 30 min.

### Flexible electrode array backend connection

Flexible printed circuit board (PCB) was designed to match the backend connection pads of MERLIN array by Eagle (Autodesk), and fabrication of flexible PCB was outsourced to PCBWay. Anisotropic conductive film adhesive (3M, catalog no. 7303-5MMX35M) was applied on the back of MERLIN array and preheated to 70°C for tacking. MERLIN array was aligned and bonded to a flexible PCB by manual flip chip die bonder (M9, Laurier) at 150°C for 30 s.

### Flexible electrode array release from wafer

MERLIN array was released from wafer by etching SiO_2_ in buffered hydrofluoric acid (Thermo Scientific Chemicals, catalog no. 044627.K2) overnight and transferred onto glass slides for further chemical modification on electrodes.

### 5A1N electrochemical polymerization

Electrochemical polymerization was performed by CV by using a PalmSens4 potentiostat (PalmSens BV). CV experiments were performed by using a three-electrode system with platinum electrodes as the working electrode, a Pt wire (CH Instruments, catalog no. CHI115) as the counter electrode, and Ag/AgCl electrode (CH Instruments, CH111) as the reference electrode. 5A1N power (TCI, catalog no. A0358) was dissolved in a PBS (VWR, catalog no. 392-0442) adjusted with hydrochloric acid (VWR, catalog no. 20246.298) to pH 1. CV electrochemical polymerization was performed in 10 mM 5A1N solution and cycled from 0.3 to 1 V (five cycles; positive direction initial sweep) at a scan rate of 10 mV/s. Electrodes were rinsed in deionized (DI) water to remove unbounded monomers.

### Fluorinated xerogel spray coating

A fluorinated sol solution was prepared by adding 7200 μl of ethanol (VWR, catalog no. 85651.320), 1260 μl of methyltrimethoxysilane (VWR, catalog no. AAAB23594-AK), 540 μl of (heptadecafluoro-1,1,2,2-tetrahydrodecyl) trimethoxysilane (Gelest, catalog no. SIH5841.5), 1960 μl of DI H_2_O (PURELAB Flex, ELGA LabWater), and 120 μl of 0.5 M HCl (VWR, catalog no. 20246.298). The solution was stirred vigorously for 1 hour. Sensors were placed on a hot plate set at 80°C. The sol solution was spray coated onto electrodes with a gravity-feed airbrush (Iwata, catalog no. N4500) pressurized at 50-psi N_2_ (Matheson gas, catalog no. NI300) for 8 s with a vertical distance of 25 cm. Sensors were removed from hot plates 2 min after spraying and allowed to dry in the air for 48 hours before testing.

### Scanning electron microscopy

Scanning electron microscopy (SEM) images were acquired using a field emission gun SEM (FEI Quanta 600). The accelerating voltage was 10 kV, and the working distance was 5 mm. All images were acquired at a high resolution of 2048 by 1768 pixels. No additional conductive coating was applied before SEM imaging. The thickness of poly-5A1N on Pt electrodes was measured by using ImageJ with *n* = 3 samples.

### Raman spectroscopy

Raman spectroscopy was performed by using LabRAM Soleil Raman Microscope (Horiba Scientific) with 532-nm excitation wavelength at a laser power of 9.2 mW. The spectra were recorded through a 50× objective, 16% neutral density filter, an acquisition time of 30 s, and 600 grating. Raman spectra were acquired from three independent samples and three randomly distributed spots per sample. Raman mapping was measured with point-by-point scanning made and mosaic image stitching features at a resolution of 100 by 100 points for an area of at least 1.6 mm by 1.6 mm with 4-s acquisition at each point. Raman mapping for 5A1N-Pt electrode was computed by MATLAB using RGB color representing relative intensity of C=C bond at 1590 cm^−1^. Raman mapping for fluorinated xerogel-5A1N-Pt electrode was computed by MATLAB using RGB color representing relative intensity of CH_3_ group at 2804 cm^−1^.

### Profilometer thickness measurement

Fluorinated xerogel thickness was measured by a profilometer (Tencor alpha step 200) over a scan distance of more than 2 mm at a speed of 5 μm/s to measure the step height of fluorinated xerogel film. Step height was acquired from three randomly distributed spots per sample and from three independent samples per batch of fabrication.

### EIS and equivalent circuit modeling

EIS was performed on Gamry R600+ potentiostat (Gamry Instruments) in a three-electrode system with NO sensing electrode, platinum wire and commercial Ag/AgCl electrode as working, counter, and reference electrode, respectively. EIS was measured at 0 V versus Ag/AgCl reference electrode with a 10-mV ac potential from 500,000 to 1 Hz in 1× PBS solution.

Custom equivalent circuit modeling was fitted on the obtained EIS spectra by using Gamry Echem Analyst software. Models were built by model editor, and data were fit by simplex method.

### CV and open-circuit potentiometry for characterizing screen-printed Ag/AgCl on-chip reference electrode

CV was performed on Gamry R600+ potentiostat (Gamry Instruments) in a three-electrode system with gold disc as working electrode, platinum wire as counter electrode, and screen-printed Ag/AgCl ink or commercial Ag/AgCl as reference electrode. CV was measured in 1 mM [Fe(CN)_6_]^3−^ in 1 M KCl solution (VWR, catalog no. BDH9258-500G) at a scan rate of 200 mV/s.

Open-circuit potentiometry was performed on Gamry R600+ potentiostat (Gamry Instruments) in a two-electrode system with painted Ag/AgCl (Creative Materials, catalog no. 126-49) as working electrode and commercial Ag/AgCl electrode (CH instrument, catalog no. CH111) as reference electrode. Open circuit potentiometry (OCP) was measured for 12 hours at a sample period of 1 s.

### Saturated NO solution preparation

A bubbling system was set up with two flasks of 1 M NaOH (VWR, catalog no. 97064-476) DI H_2_O (PURELAB Flex, ELGA LabWater), 1× PBS (0.01 M, pH 7.4) (VWR, catalog no. 392-0442), and DI H_2_O (PURELAB Flex, ELGA LabWater) in a well-ventilated chemical hood (fig. S8A). The system was purged with ultrahigh purity nitrogen gas (Matheson, catalog no. G1959175) for 1 hour to remove oxygen gas. NO gas (9.5%; Matheson gas, catalog no. G2659782) was bubbled for 1 hour to reach a saturated concentration of 200 μM at 0°C measured by commercial NO probe (World Precision Instrument). Saturated NO solutions were freshly made each day.

### Determination of NO oxidation potential by LSV

Linear scan voltammetry (LSV) was performed by using PalmSens4 potentiostat (PalmSens BV). LSV experiments were performed by using a two-electrode system with NO sensing electrode as the working electrode and on-chip screen-printed Ag/AgCl (Creative Materials, catalog no. 126-49) as the reference and counter electrode. NO sensing electrode was immersed with 25 μM NO in PBS (0.01 M, pH 7.4) (VWR, catalog no. 392-0442). LSV peak was fitted by using linear baseline subtraction in the PSTrace software.

### MERLIN array in vitro sensing calibration

Sensor calibration was performed in a mechanically stirred, deoxygenated PBS solution. Electrochemical interference solutions of nitrite (Sigma-Aldrich, catalog no. S2252-500G), ascorbic acid (Sigma-Aldrich, catalog no. A4544-25G), and uric acid (Sigma-Aldrich, catalog no. U0881-10G) were added to reach a concentration of 500, 100, and 100 μM, respectively, before adding the saturated NO solutions. Ten aliquots of saturated NO solution were added to reach a cumulative NO concentration 50 nM, 100 nM, 250 nM, 500 nM, 750 nM, 1 μM, 1.5 μM, 2 μM, 4 μM, and 6 μM (fig. S8B). A linear regression of peak current to each aliquot of NO solution against the cumulative NO concentration was plotted. The slope of the linear regression was the sensitivity of NO sensing electrode. The LOD is calculated by three times SD divided by the sensitivity of the electrode. The selectivity of each electrode is calculated by the logarithmic ratio of sensitivity toward NO and sensitivity toward electrochemical interferences. Sensitivity, selectivity, and LOD are defined based on the following equations

Sensitivity(SNO)=ΔIΔ[NO] (3)

Log10(SelectivityNO,X)=log10SNOSX (4)

Limit of detection(LOD)=3σSNO (5)

### MERLIN array in vitro sensing calibration in SWF

SWF composition consists of 110 mM sodium chloride (Sigma-Aldrich, catalog no. S9888-25G), 2.2 mM calcium chloride (Sigma-Aldrich, catalog no. 383147-100G), 2.7 mM potassium chloride (Sigma-Aldrich, catalog no. P3911-25G), 0.5 mM magnesium chloride (Sigma-Aldrich, catalog no. M8266-100G), bovine serum albumin (34 g/liter; Sigma-Aldrich, catalog no. 126575-10GM), 1.3 mM potassium phosphate (Sigma-Aldrich, catalog no. P0662-25G), and 20 mM sodium bicarbonate (Sigma-Aldrich, catalog no. S6014-25G) (*68*). The addition of electrochemical interference, saturated NO solution, and calculation of sensor performance was the same as calibration in PBS.

### Rat skin wound model NO sensing in vivo

All procedures were approved by the University of Pittsburgh Institutional Animal Care and Use Committee (IACUC) and the DoD Animal Care and Use Review Office (IACUC protocol number 22091435) and were carried out in accordance with the approved guidelines.

Rats were induced and maintained under anesthesia using 2% isoflurane, and the surgical site was shaved and prepared in standard surgical fashion. Two 2.0-cm-diameter, full-thickness skin wounds were made by sharp incision on either side of the midline in the dorsum of 250 to 300 g of male Sprague-Dawley Rats (Charles River, Wilmington, MA). A 2.0-cm–inner diameter silicone wound splint (Grace Bio-labs, Bend, OR) was then affixed to the wound margins using 4-0 nylon sutures to prevent wound contraction. The wounds were then covered with a Tegaderm Transparent Film Dressing (3M, catalog no. 7100252805).

NO measurement was performed on days 1, 3, 5, and 7 for a duration of 1 hour on the wound. Sensors were sterilized by 70% ethanol and 30 min of UV light. MERLIN arrays were polarized in 1× PBS (VWR, catalog no. 392-0442) for 15 min before placing on wound. Twenty-five microliters of 10 mM l-arginine (Sigma Alrich, catalog no. A5006-100G) was added to stimulate NO production by iNOS followed by 50 μl of 1× PBS (VWR, catalog no. 392-0442) added as a control. Exponential fitting with equation $\text{current}=a+b^{*}\text{exp}\left(c^{*}\text{time}\right)$ was performed with 30 min of chronoamperometry data. The baseline current is plotted against time for continuous sensing. Concentration of NO was converted by a 10-min average baseline current divided by sensitivity.

In a separate cohort of *n* = 5 rats as the control group, two 2.0-cm-diameter, full-thickness skin wounds were made by sharp incision the same way as previously described. Wounds were affixed to the wound margins using 4-0 nylon sutures to prevent wound contraction and were then covered with a Tegaderm Transparent Film Dressing (3M, catalog no. 7100252805). All rats were euthanized on day 7 post-wounding, and the wound tissue was excised and fixed in 10% neutral-buffered formalin for histologic analysis.

### MERLIN arrays NO measurement in vivo

MERLIN arrays were lifted from glass slides by using sharp tweezers. After placing the sensor on the rat skin wound, backend of flexible PCB was connected to a custom breakout PCB (PCBWay) interfacing with PalmSens 4 (PalmSens BV) connected with MUX8-R2 multiplexer (PalmSens BV) for eight-channel measurement. Sensor array measurement was performed by using method script on PSTrace with 2-s interval per electrode for a continuous measurement of 60 min per session.

### Histological analysis and quantification

At the time of euthanasia, wound tissue was excised using a 6-mm biopsy punch and fixed in 10% neutral-buffered formalin. The tissues were then processed and embedded in paraffin before sectioning at 5 μm and staining with H&E. H&E-stained slides were then imaged at ×40 magnification on a whole slide imager (Motic EasyScan, Motic Digital Pathology). High-resolution H&E-stained tissue sections were imported into QuPath (version 0.4.3) for manual and automated annotation-based measurements. All measurements were made in a blinded fashion by a trained investigator to avoid bias. The number of cells detected within each section was normalized to the area of the section and is reported as cells/mm^2^.

Cell densities were measured within the granulation tissue of both experimental (wounds with NO sensor measurement) and control (de novo wounds with no sensor measurement) groups. We used QuPath’s cell detection algorithm to identify the nuclei on the basis of their hematoxylin intensity and morphological characteristics such as size and shape. The cell density was then calculated using the following formula: Cell density = total number of nuclei in the region of interest (ROI)/total area of the ROI (square millimeters).

For quantifying thickness of epithelium and granulation tissue, we manually delineated the epithelium and granulation tissue areas on each biopsied section to annotate the ROIs. The total area as well as the width of epithelium and granulation tissue were calculated using QuPath’s measurement tools. The thickness was then calculated by dividing the total area with respective width of the tissue section. For consistency and uniformity across samples, the same algorithm and detection parameters were used throughout the measurements.

The mean values for epithelial and granulation tissue thickness, as well as the cell density, were compared between the experimental and control groups. Statistical analysis was performed using the analysis of variance (ANOVA) test, with *P* < 0.05 considered statistically significant.

### Biocompatibility test

Biocompatibility testing compliant with ISO 10993-1:2018 Biological Evaluation of Medical Devices was completed by North American Science Associates (NAMSA) (670 Wales Road, Northwood, OH 43619, USA) and covered the five ISO and FDA-recommended biocompatibility endpoints described in ISO 10993-1:2020, including cytotoxicity, sensitization, intracutaneous irritation, acute systemic toxicity, and pyrogenicity.

## Supplementary Materials

### This PDF file includes:

Supplementary Note Figs. S1 to S19 Tables S1 to S3 References
