# Supplementary material for: Wound state monitoring by multiplexed, electrochemical, real-time, localized, inflammation-tracking nitric oxide sensor (MERLIN)
Source: Sci Adv. 2025 May 28;11(22):eadv2385. doi: 10.1126/sciadv.adv2385 (PMC12118596; doi:10.1126/sciadv.adv2385)
Supplement: Supplementary file 1 — Supplementary Note Figs. S1 to S19 Tables S1 to S3 References [file sciadv.adv2385_sm.pdf]

Science Advances
AAAS

Supplementary Materials for

Wound state monitoring by multiplexed, electrochemical, real-time, localized, inflammation-tracking nitric oxide sensor (MERLIN)

Liyang Wang *et al.*

Corresponding author: Tzahi Cohen-Karni, tzahi@andrew.cmu.edu

Sci. Adv. **11**, eadv2385 (2025)
DOI: 10.1126/sciadv.adv2385

This PDF file includes:

Supplementary Note
Figs. S1 to S19
Tables S1 to S3
References

Notes

Constant phase element modelling to fit for non-ideal double layer capacitance at the electrode-electrolyte interface

Poly-5A1N modified electrodes and fluorinated xerogel spray-coated electrodes contain an additional capacitive and resistive element related to successive polymeric layer deposition. Due to non-ideal conditions, capacitive elements are modeled as constant phase elements defined by

$$Z_{DL} = \frac{1}{Q_{DL}(i\omega^{\alpha})} \quad (1)$$

where Z_{DL}, Q_{DL}, ω and α are constant phase element impedance, constant phase element capacitance, angular frequency of voltage perturbation and constant phase.(*62, 63*)

MERLIN mechanics analytical calculations (*80*)

Model: 100 nm thick Pt, 10 μm thick SU8, 3 μm thick fluorinated xerogel for Fig. S7

The distance between the neutral axis and bottom of the thin film is y0:

$$y_0 = \frac{h_{SU8}}{2} \cdot \frac{1 + \frac{2h'_{SU8} + h_m}{h_{SU8}}\left(\frac{E_{Pt}}{E_{SU8}} - 1\right)\frac{nb_m}{bh_{SU8}} - \frac{E_{xerogel}h_{xerogel}}{E_{SU8}h_{SU8}^2}}{1 + \left(\frac{E_{Pt}}{E_{SU8}} - 1\right)\frac{nb_m}{bh_{SU8}} + \frac{E_{xerogel}h_{xerogel}}{E_{SU8}h_{SU8}}}$$

Where h_{SU8} is height of SU-8, h'_{SU8} is first layer of SU-8, h_m is height of metal stack, E_{Pt} is Young's modulus of Pt, E_{SU8} is the Young's modulus of SU-8, n is number of metal traces, b_m is the width of metal, b is the width of SU-8, $E_{xerogel}$ is Young's modulus of xerogel and $h_{xerogel}$ is height of xerogel.

$h_{SU8} = 10\mu m,\ h'_{SU8} = 5\mu m,\ h_m = 0.1\mu m,\ E_{Pt} = 172GPa,$(*81*) $E_{SU8} = 2.3GPa,$(*82, 83*) $n = 6,\ b_m = 1500\mu m,\ b = 11100\ \mu m,\ E_{xerogel} = 12GPa,$(*84*) $h_{xerogel} = 3\mu m.$

$$y_0 = 1.79\ \mu m$$

Bending stiffness of the thin film is:

$$EI = E_{SU8}\, bh_{SU8}\left(\frac{1}{3}h_{SU8}^2 - h_{SU8}y_0 + y_0^2\right) + E_{xerogel}bh_{xerogel}\left(\frac{1}{3}h_{xerogel}^2 + h_{xerogel}y_0 + y_0^2\right) + (E_{Pt} - E_{SU8})nh_m b_m[\frac{1}{3}h_m^2 + h_m(h'_{SU8} - y_0) + (h'_{SU8} - y_0)^2$$

EI = 1.09 E+7 GPa μm^4

For the wrap state to be energetically favorable,

$\gamma \geq \gamma_c = \frac{EI}{2R^2b}$

Where R = 1 cm

$$\gamma_c = 4.91\ mJm^{-2}$$

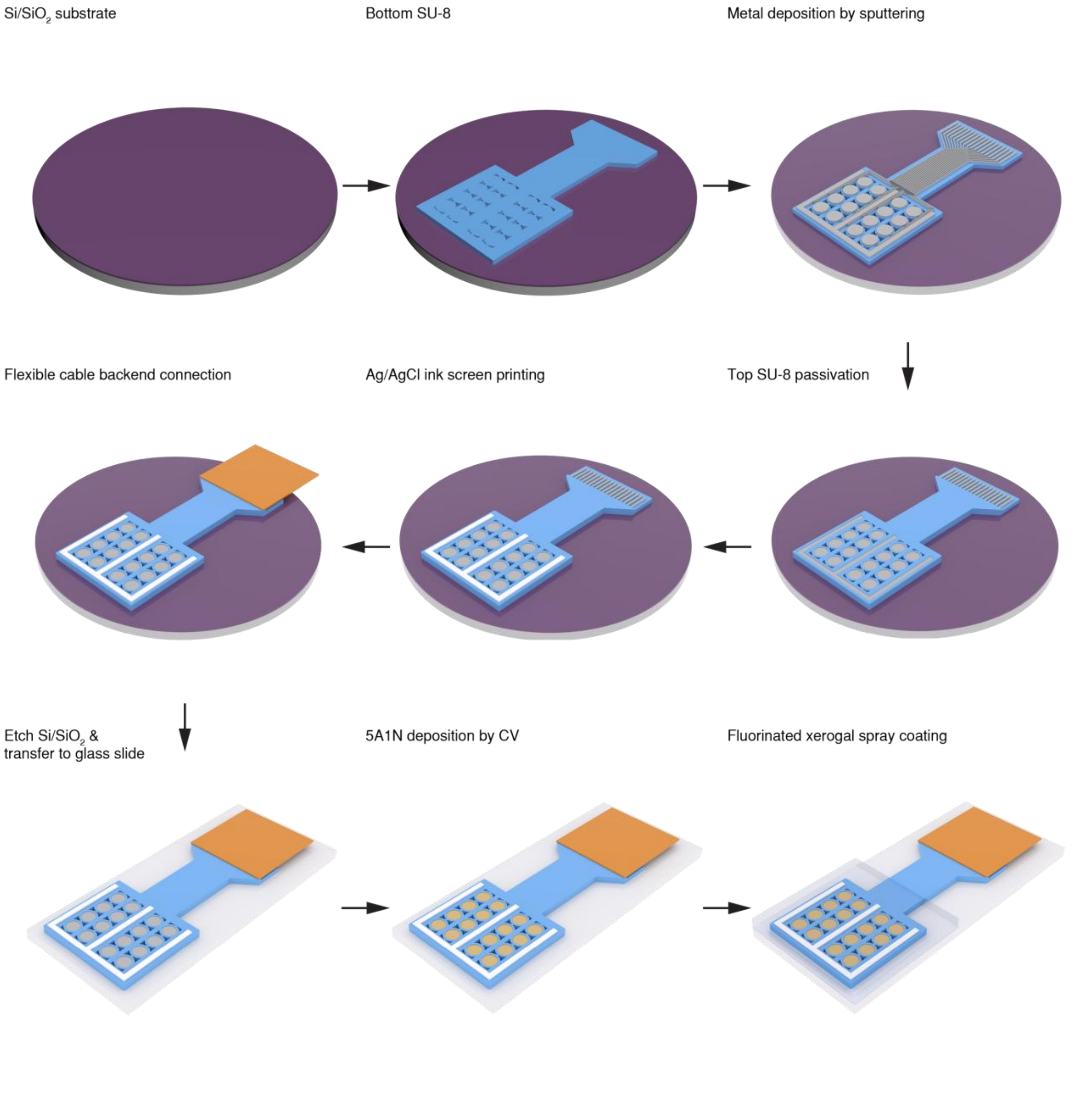

Figure S1. MERLIN fabrication process flow. MERLIN arrays were fabricated on Si/600nm SiO_2 wafers. 5 μm SU-8 was patterned by spin coating and photobiography. 20 μm Cr and 80 μm Pt was deposited by sputtering, followed by 5 μm SU-8 passivation. Ag/AgCl ink was screen printed as the on-chip reference electrode, followed by flexible PCB connection onto flexible array by ACF adhesives. Flexible arrays were released by etching $SiSiO_2$ in BHF. 5A1N selective layer was deposited by electrochemical polymerization and fluorinated xerogel was deposited by spray coating.

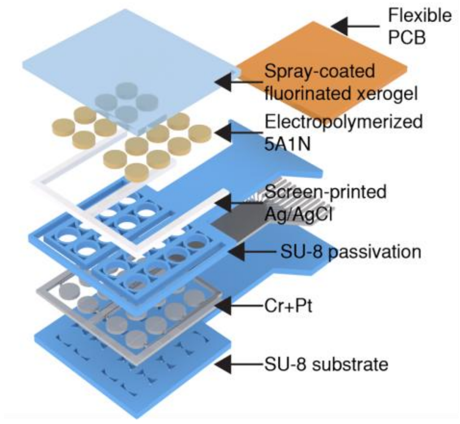

Figure S2. Expanded view of MERLIN.

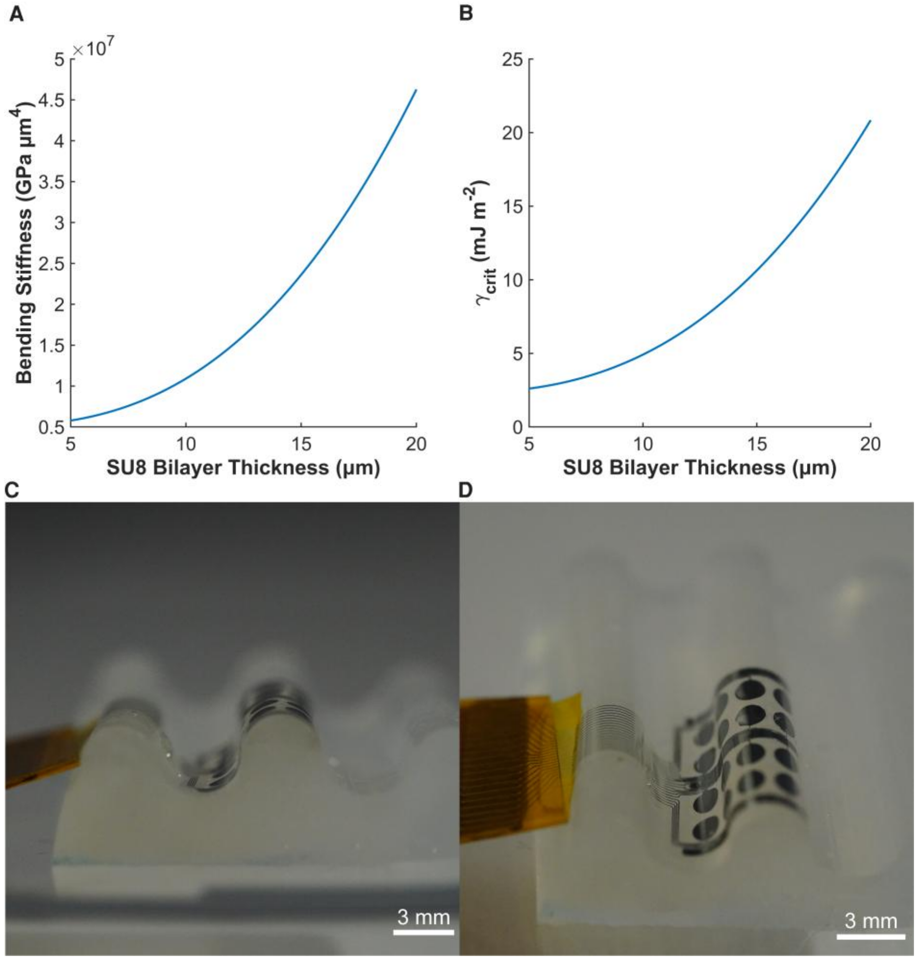

Figure S3. MERLIN's mechanical flexibility allows conformal contact to wounds. (**A**) Bending stiffness analytical calculation based on MERLIN materials and geometry. (**B**) Adhesive energy per unit area calculation based on MERLIN materials and geometry. (**C**) Side view image of MERLIN on 3 mm diameter grooves of the phantom tissue. (**D**) 45 degree view image of MERLIN on 3 mm diameter grooves of the phantom tissue.

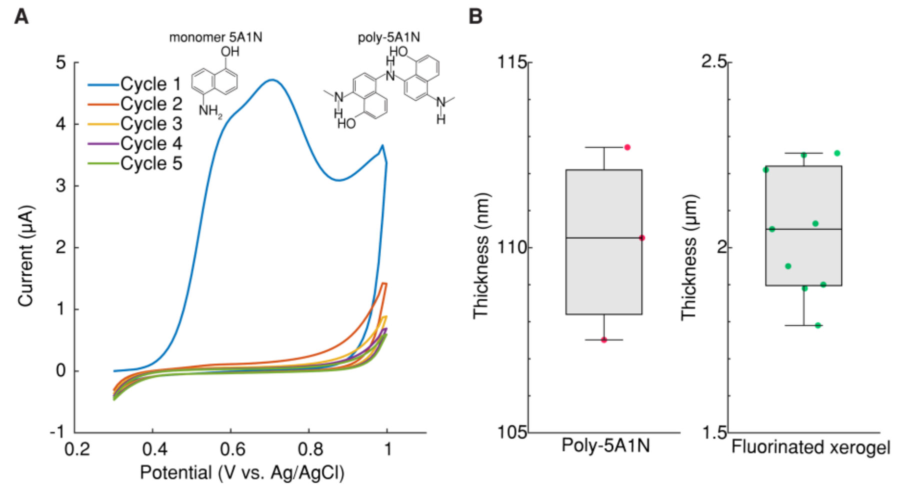

Figure S4. Selective layer deposition and thickness measurement. (**A**) Electrochemical polymerization of 5-amino-1-naphthol (5A1N) by cyclic voltammetry. Insert: monomer 5A1N and poly-5A1N. (**B**) Thickness measurement of electrodeposited 5A1N by SEM and fluorinated xerogel by profilometer, n = 3 and n = 9 for poly-5A1N and fluorinated xerogel respectively.

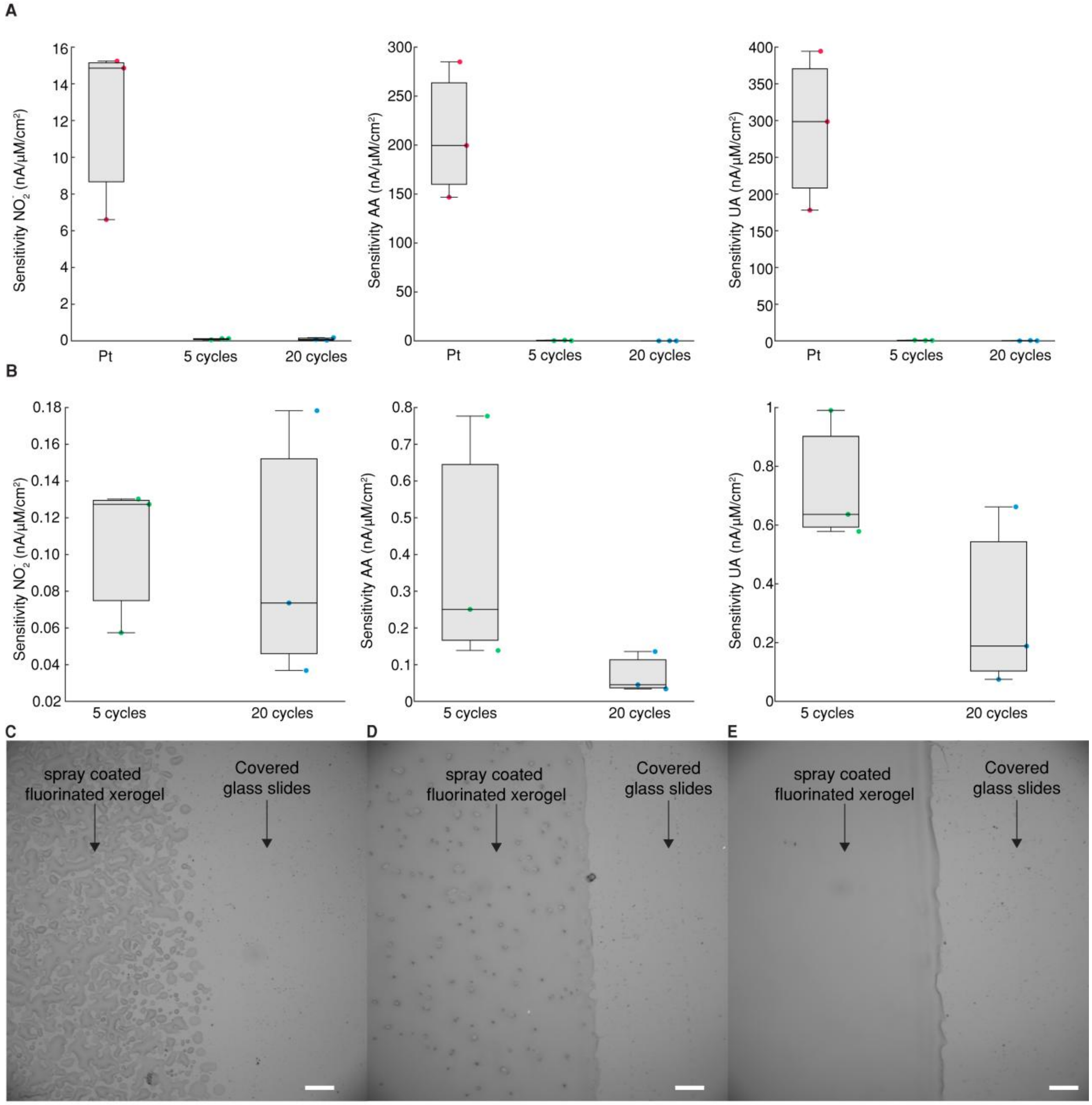

Figure S5. Optimization of selective layer deposition. (**A**) Plot of sensitivity against electrochemical interference such as nitrite, ascorbic acid, and uric acid for plain platinum (Pt), 5 cycles of 5A1N cyclic voltammetry electrochemical polymerization (5 cycles), and 10 cycles of 5A1N cyclic voltammetry electrochemical polymerization (10 cycles). (n = 3) (**B**) Zoomed in plot of sensitivity against electrochemical interference such as nitrite, ascorbic acid, and uric acid for 5 cycles of 5A1N cyclic voltammetry electrochemical polymerization (5 cycles), and 10 cycles of 5A1N cyclic voltammetry electrochemical polymerization (10 cycles). (n = 3) (**C**) Image of spray coated fluorinated xerogel for 5 seconds, (**D**) for 8 seconds and (**E**) for 10 seconds to achieve a homogenous layer. Scale bar 100 µm.

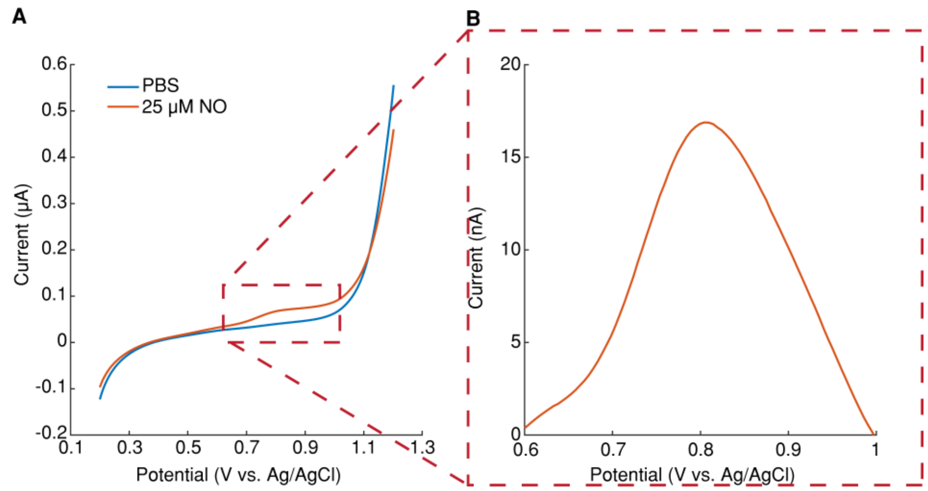

Figure S6. Chronoamperometry (CA) operation potential characterization by linear scan voltammetry (LSV). (**A**) Determination of NO oxidation peak at 0.81V by LSV. (**B**) Zoomed-in view of baseline subtracted LSV curve with 25 μM NO. LSV parameters: 0.2V-1.2V, 10mV/s.

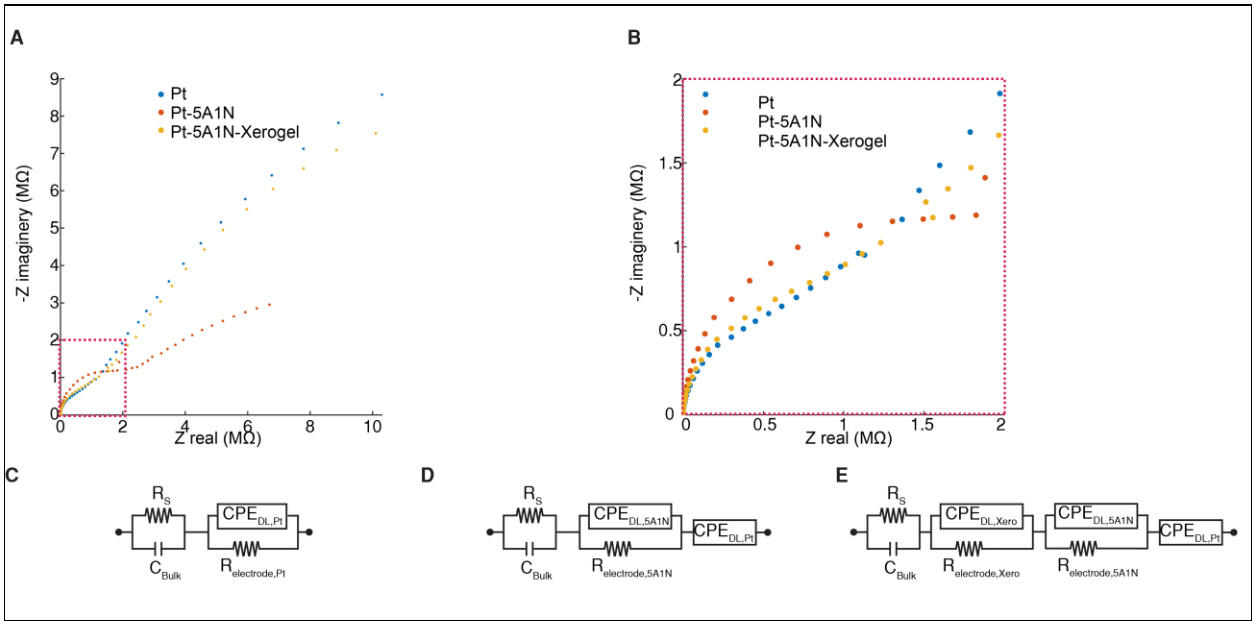

Figure S7. EIS Nyquist plot and equivalent circuit model for electrode-electrolyte interfaces. (**A**) Nyquist plot of electrochemical impedance of MERLIN electrodes post chemical modifications. (**B**) Zoomed in Nyquist plot of electrochemical impedance of MERLIN electrodes. (**C**) Equivalent circuit model for platinum electrode. (**D**) Equivalent circuit model for 5A1N modified platinum electrode. (**E**) Equivalent circuit model for fluorinated xerogel-5A1N-platinum electrode.

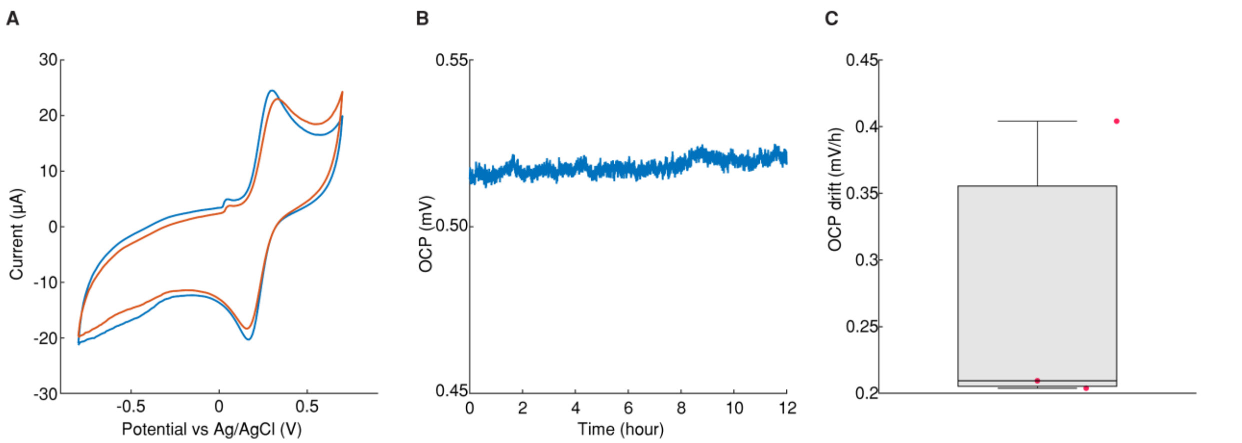

Figure S8. On-chip reference electrode characterization. (**A**) Cyclic voltammetry (CV) characterization comparison by using gold disc electrode as working electrode, platinum wire as counter electrode and screen-printed Ag/AgCl ink and commercial Ag/AgCl as reference electrode in 1M $[Fe(CN)_6]^{3-}$ solution. Blue – Ag/AgCl ink as reference electrode. Orange – commercial Ag/AgCl as reference electrode. (**B**) Representative long-term stability of screen-printed Ag/AgCl ink by open circuit potentiometry (OCP) measurement against commercial Ag/AgCl in 1xPBS over 12 hours. (**C**) Rate of OCP drift over 12 hour period (n = 3).

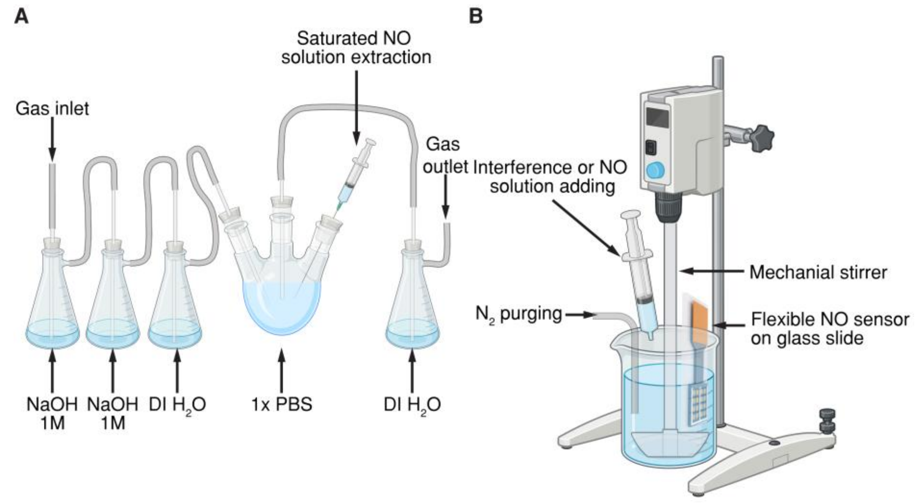

Figure S9. MERLIN *in vitro* calibration schematic illustration. (**A**) NO saturated solution preparation by bubbling NO gas through 1 M NaOH, DI water and into 1xPBS in ice bath. (**B**) *In vitro* setup for NO sensor sensitivity and selectivity calibration.

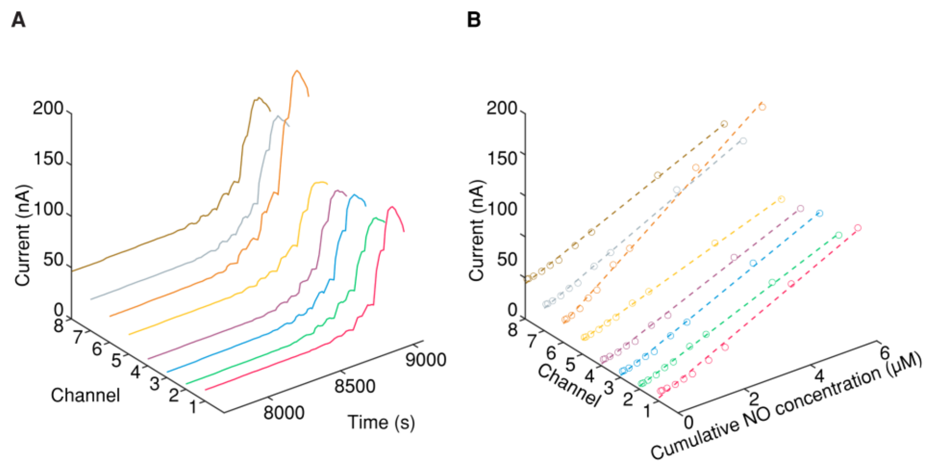

Figure S10. Representative 8-channel multiplexed MERLIN array calibration. (**A**) Representative 8-channel multiplexed current measurement during calibration. (**B**) Representative 8-channel calibration curve and linear regression of current against cumulative NO concentration.

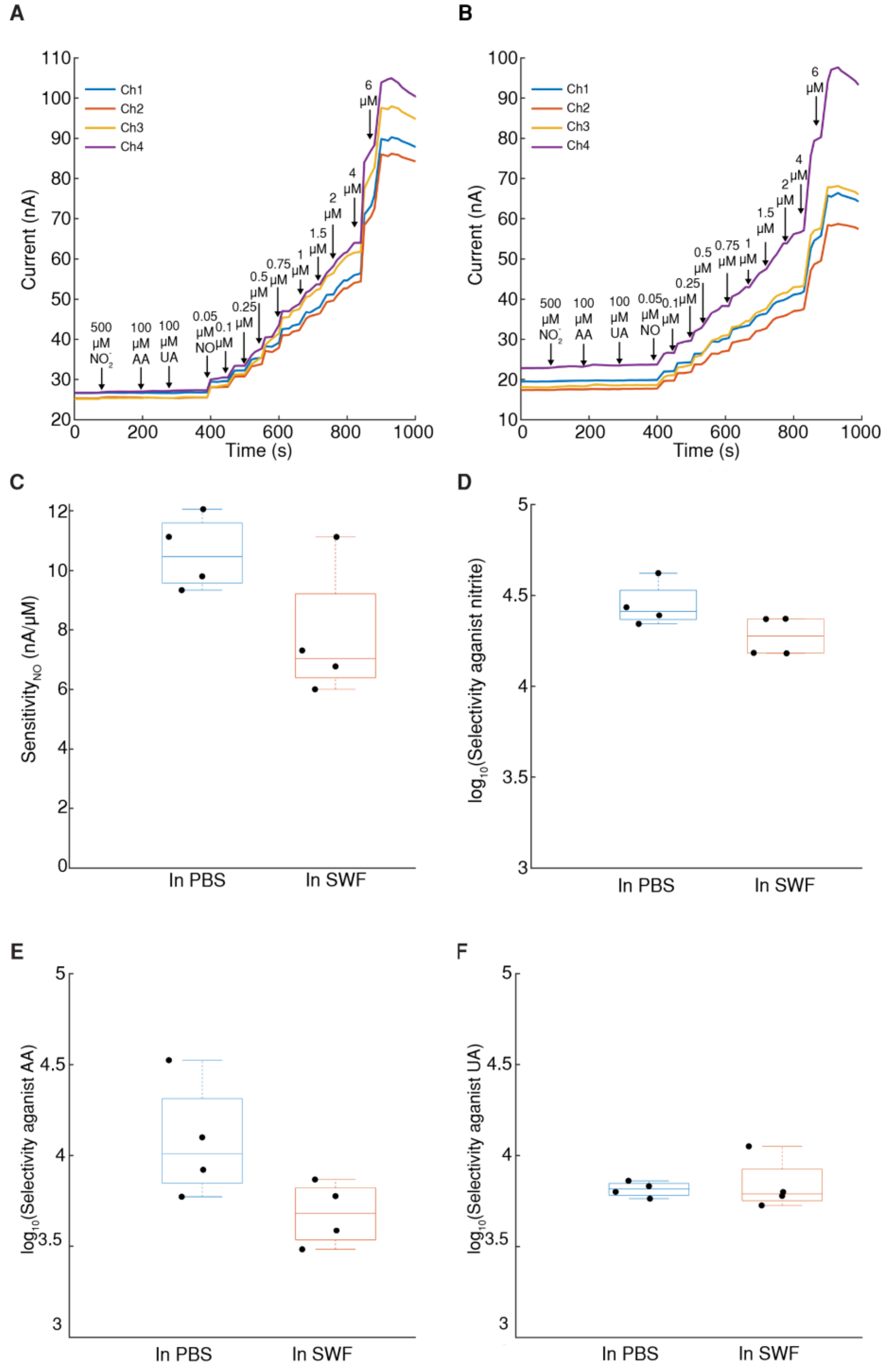

Figure S11. MERLIN NO sensing calibration comparison in phosphate buffer solution (PBS) and simulated wound fluid (SWF). (**A**) Representative multiplexed NO sensor calibration in PBS. (**B**) Representative multiplexed NO sensor calibration in SWF. (**C**) MERLIN NO sensitivity comparison in PBS vs SWF. (**D**) MERLIN nitrite selectivity comparison in PBS vs SWF. (**E**) MERLIN AA selectivity comparison in PBS vs SWF. (**F**) MERLIN UA selectivity comparison in PBS vs SWF. No statistical significance observed for NO sensitivity, selectivity against nitrite, AA and UA ($P > 0.05$ by ANOVA).

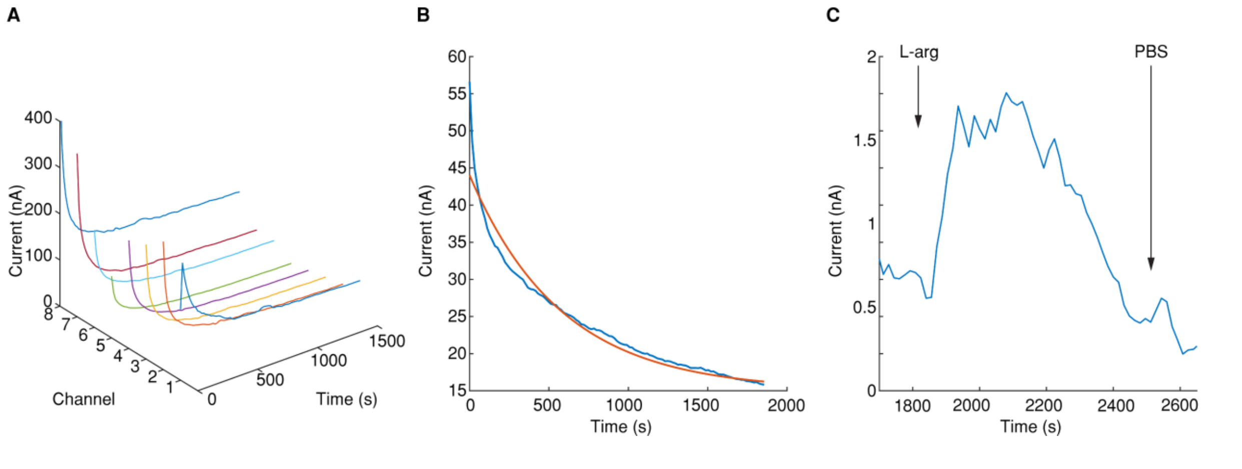

Figure S12. Extrapolation of basal current and MERLIN function validation. (**A**) Representative 8-channel multiplexed NO sensing *in vivo*. (**B**) Representative exponential fitting to determine baseline current and baseline NO concentration. Blue – raw data collected by MERLIN. Orange – exponentially fit data. (**C**) Addition of L-arg induces production of nitric oxide and validates functionality of MERLIN array. Addition of PBS was added as control.

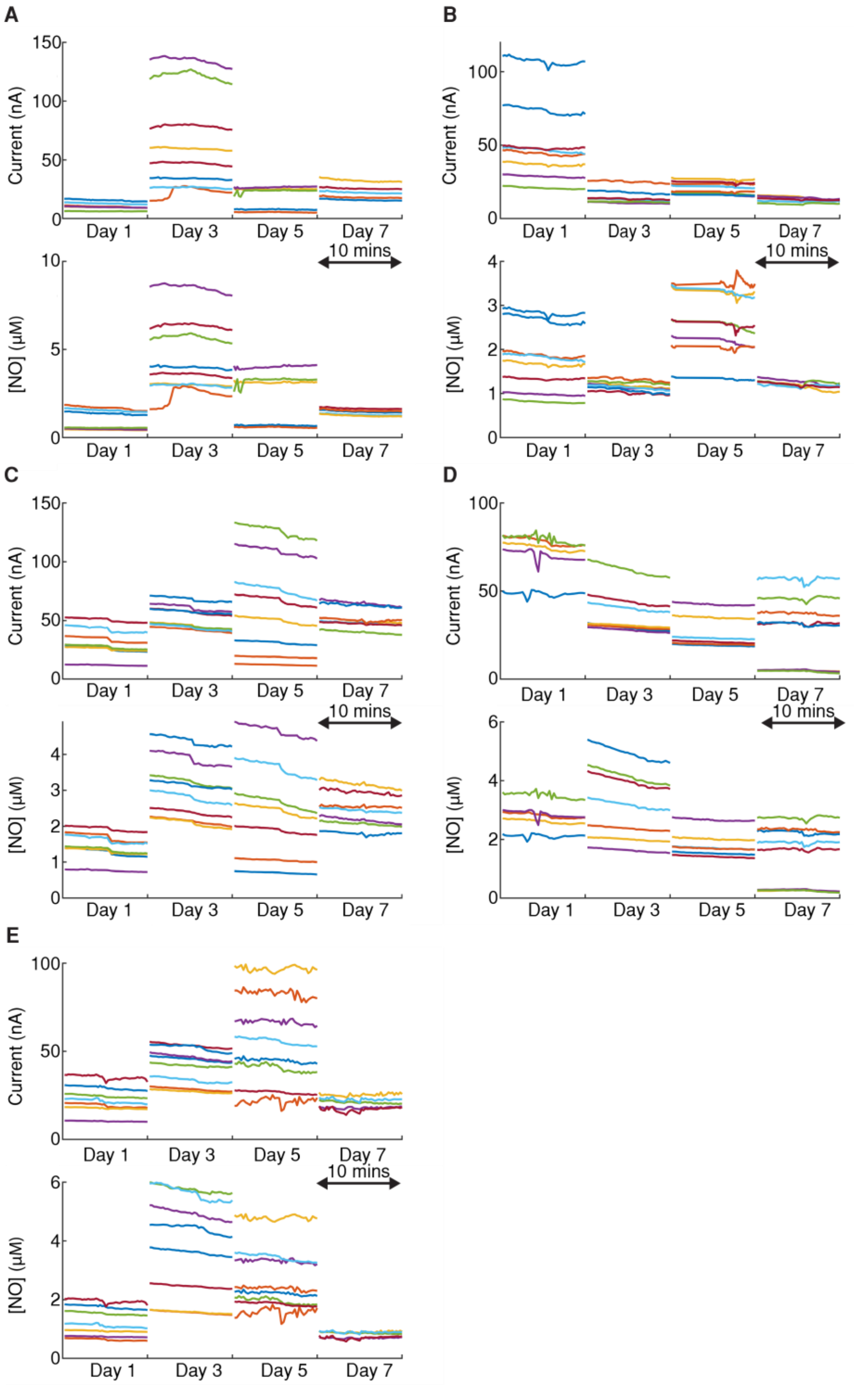

Figure S13. Continuous NO measurement on rat skin wound *in vivo*. Continuous current measurement and converted NO concentration measurement on (**A**) rat 1 right wound, (**B**) rat 2 left wound, (**C**) rat 3 right wound, (**D**) rat 4 left wound, and (**E**) rat 4 right wound.

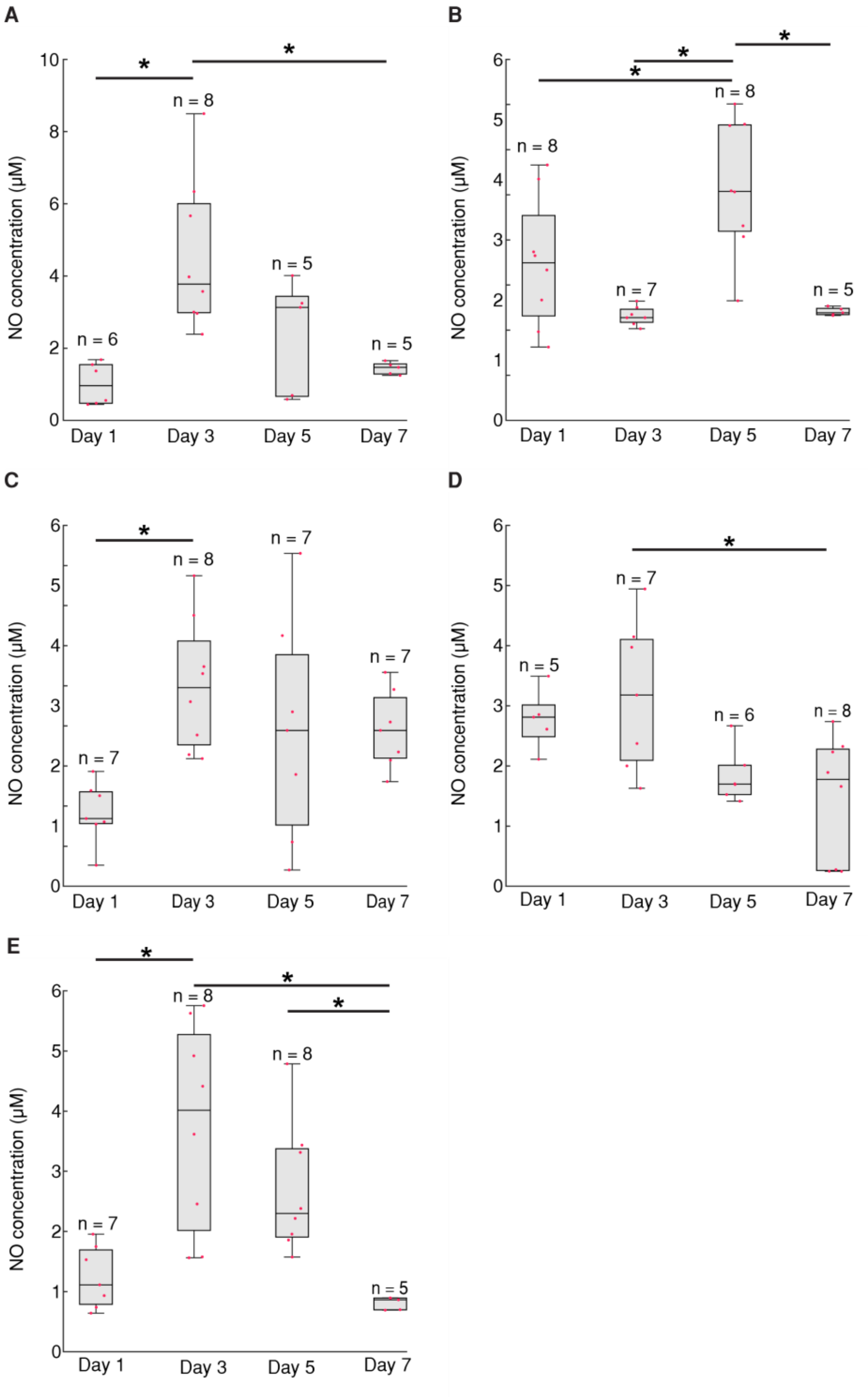

Figure S14. Temporal NO concentration summary measured by MERLIN. Boxplot of NO concentration measured by MERLIN on (**A**) rat 1 right wound, (**B**) rat 2 left wound, (**C**) rat 3 right wound, (**D**) rat 4 left wound, (**E**) rat 5 right wound. * $P < 0.05$

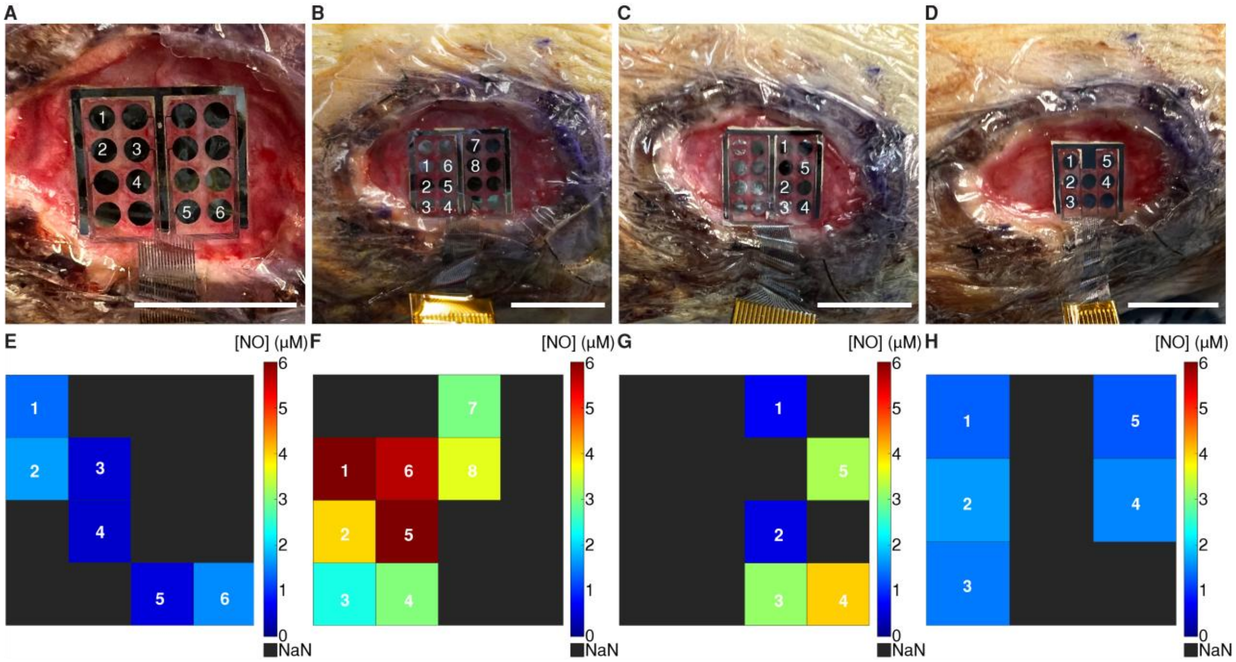

Figure S15. Spatial NO measurement by MERLIN on rat 1 right wound. (**A**, **B**, **C**, **D**) MERLIN array placement on rat 1 right wound on Day 1, 3, 5, and 7. Scale bar, 1 cm. (**E**, **F**, **G**, **H**) NO concentration mapping readout by MERLIN on Day 1, 3, 5, and 7. Color bar in units of μM NO.

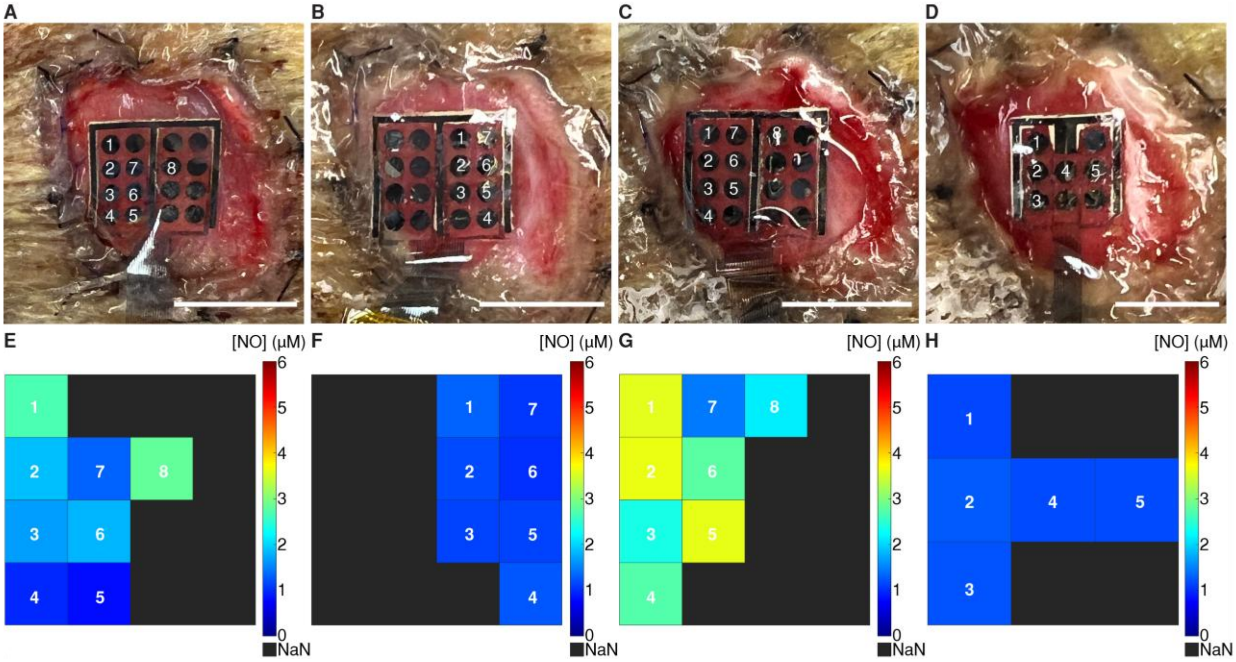

Figure S16. Spatial NO measurement by MERLIN on rat 2 left wound. (**A**, **B**, **C**, **D**) MERLIN array placement on rat 2 left wound on Day 1, 3, 5, and 7 post surgery. Scale bar, 1 cm. (**E**, **F**, **G**, **H**) NO concentration mapping readout by MERLIN on Day 1, 3, 5, and 7. Color bar in units of μM NO.

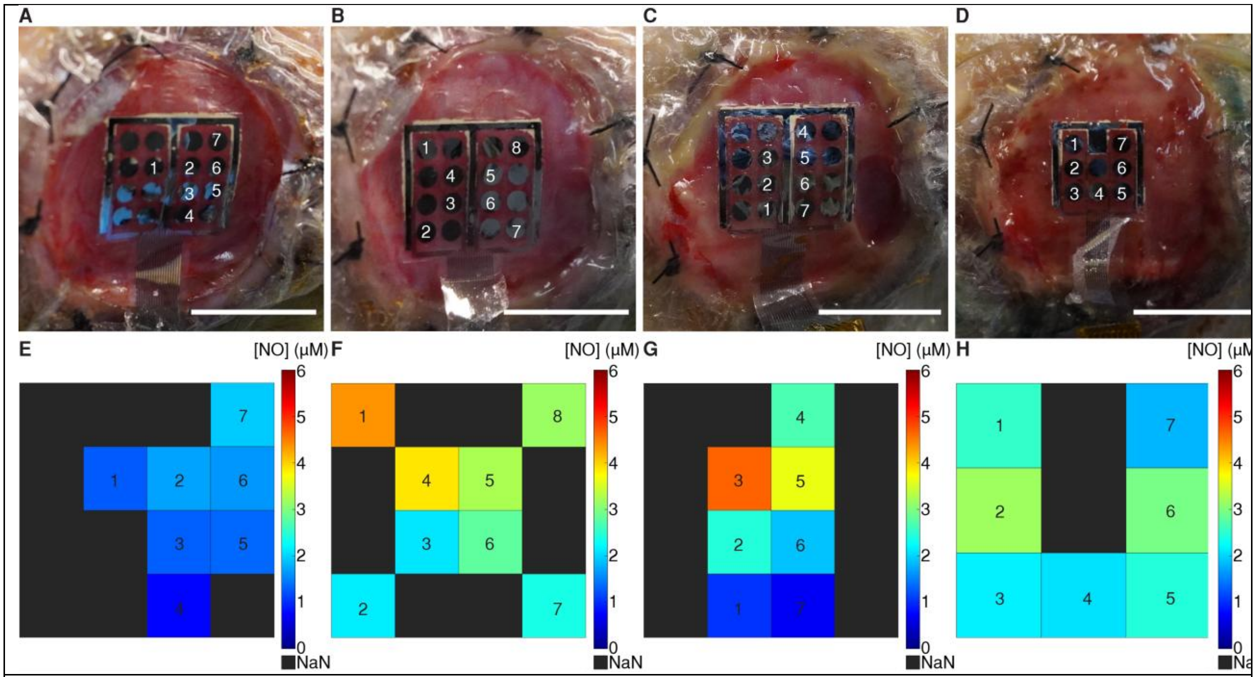

Figure S17. Spatial NO measurement by MERLIN on rat 3 right wound. (A, B, C, D) MERLIN array placement on rat 3 right wound on Day 1, 3, 5, and 7 post surgery. Scale bar, 1 cm. (**E**, **F**, **G**, **H**) NO concentration mapping readout by MERLIN on Day 1, 3, 5, and 7. Color bar in units of μM NO.

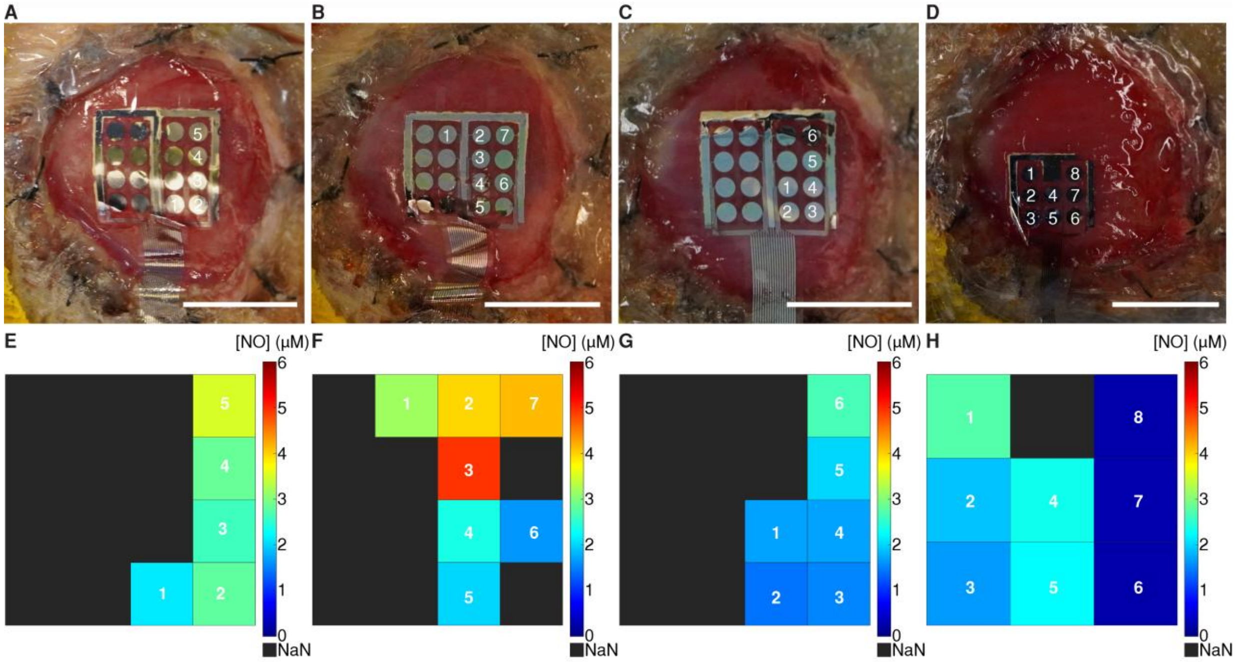

Figure S18. Spatial NO measurement by MERLIN on rat 4 left wound. (**A**, **B**, **C**, **D**) MERLIN array placement on rat 4 left wound on Day 1, 3, 5, and 7 post surgery. Scale bar, 1 cm. (**E**, **F**, **G**, **H**) NO concentration mapping readout by MERLIN on Day 1, 3, 5, and 7. Color bar in units of µM NO.

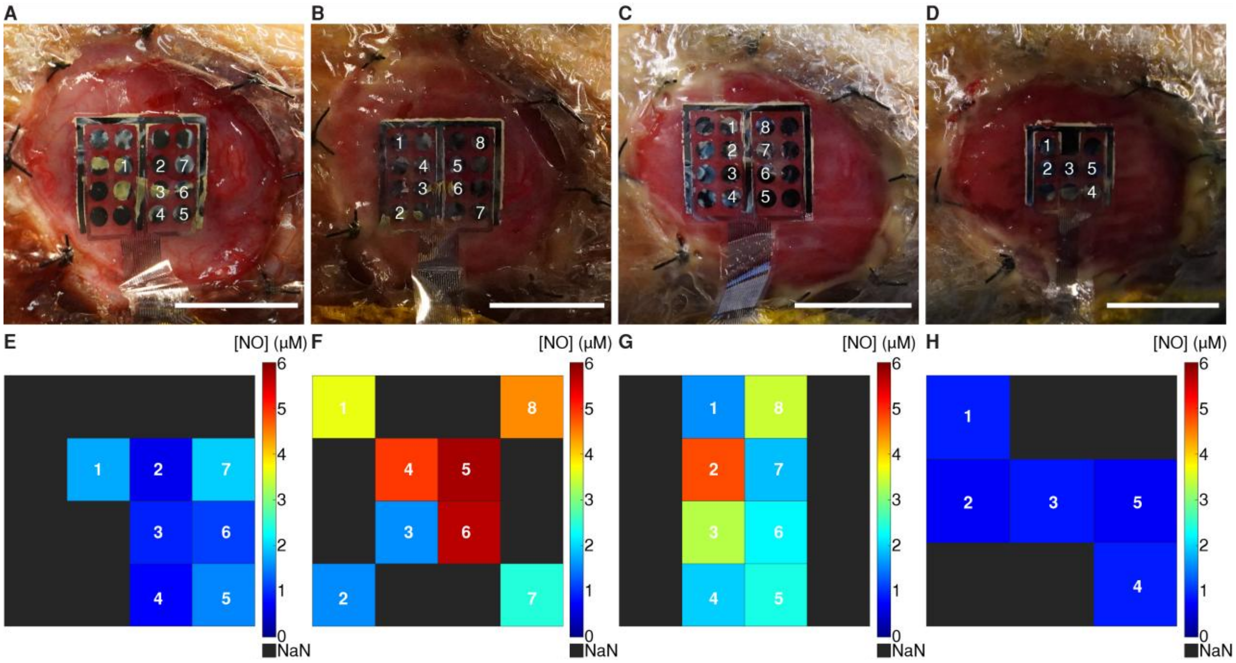

Figure S19. Spatial NO measurement by MERLIN on rat 4 right wound. (**A**, **B**, **C**, **D**) MERLIN array placement on rat 4 left wound on Day 1, 3, 5, 7 post surgery. Scale bar, 1 cm. (**E**, **F**, **G**, **H**) NO concentration mapping readout by MERLIN on Day 1, 3, 5, 7. Color bar in units of μM NO.

Structure	Sensitivity per unit area	Linear Range	LOD	Selectivity	Reproducibility	Sensor application	Ref
Rigid carbon fiber<br><br>Nafion - polymeric porphyrin - carbon fiber	Not reported	Up to 300 μM	Not reported	Not reported	Not reported	*In vitro* measurement from endothelial and muscle cells	*(49)*
Rigid wire<br><br>Hemin / carbon nanotubes / chitosan - carbon fiber electrode	Not reported	0.25-1uM	25nM	Not reported	n = 16	Rat brain *in vivo* (n = 1 rat data presented, at 1 time point)	*(85)*
Rigid field effect transistor<br><br>Hemin – graphene	54 μS/1,000 nM	Not reported	0.3 nM	Not reported	Not reported	*In vitro* experiment with stimulated macrophages	*(86)*
Rigid wire<br><br>Fluorinated xerogel – Pt black	32.9 $nA/\mu M/cm^2$	Up to 0.3uM	6 nM	Not reported	n = 6	Needle probe, rat brain *in vivo* (n = 1 rat data presented, at 1 time point)	*(87)*
Rigid wire<br><br>Fluorinated xerogel – platinized Pt	11.7 $nA/\mu M/cm^2$	Up to 3 μM	9.55 nM	Not reported	n = 10	rat brain *in vivo* (n = 5 rats, at 1 time point)	*(88)*
Rigid wire<br><br>Fluorinated xerogel – 5A1N - Pt	395 $nA/\mu M/cm^2$	0.01 - 6 μM	1 nM	$k\_NO_2^-$ = 4.9 ± 0.5<br>k_AA = 3.65 ± 0.65	n ≥ 8	*In vitro* experiment with stimulated macrophages	*(43)*
Flexible carbon ultramicroelectrode arrays<br><br>Pyrolyzed photoresist	Not quantified	1 – 100 μM	20 nM	Not reported	Not reported	in simulated wound media	*(89)*
Flexible single electrode<br><br>Poly(eugenol)- Au on PLLA-PTMC	26.45 $nA/\mu M/cm^2$ (0 - 5uM)	10nM – 100μM	3.92 nM	Response current ratio < 15%	n =3	rabbit joint cavity *in vivo* (n = 3 rabbits, at 5 time points)	*(40)*
Flexible single channel organic electrochemical transistor<br><br>5A1N – Au on polyimide	94 mV/dec	3 nM to 100 μM	3 nM	Not reported	Not reported	Rabbit joint cavity *in vivo* (n = 3)	*(41)*
**Flexible 4 x 4 electrode array**<br><br>**fluorinated xerogel - 5A1N – Pt on SU-8**	**883 ± 283** $nA/\mu M/cm^2$	**up to 6 μM**	**8.00 nM**	**$k\_NO_2^-$ =4.44 ± 0.43**<br>**K_AA =3.58 ± 0.44**<br>**K_UA =3.84 ± 0.45**	**n = 343**	**Rat skin wound in vivo (n = 4 rats, measured at 4 time points per animal)**	**Our work**

Table S1. Comparison among state-of-the-art NO electrochemical sensors to MERLIN.

	Rs (ohm)	$C_{DL,Pt}$ ($S\text{-}s^{\alpha}$)	$C_{DL,5A1N}$ ($S\text{-}s^{\alpha}$)	$C_{DL,Xero}$ ($S\text{-}s^{\alpha}$)	X^2
Pt (n= 19)	$1.14 \times 10^3 \pm 2.17 \times 10^2$	$2.68 \times 10^{-6} \pm 1.41 \times 10^{-7}$	-	-	5.63×10^{-4}
5A1N (n= 8)	$7.97 \times 10^2 \pm 2.40 \times 10^2$	$4.67 \times 10^{-7} \pm 2.39 \times 10^{-7}$	$5.69 \times 10^{-8} \pm 1.63 \times 10^{-8}$	-	7.86×10^{-3}
Xerogel (n= 8)	$5.86 \times 10^5 \pm 1.88 \times 10^5$	$7.32 \times 10^{-8} \pm 4.32 \times 10^{-8}$	$1.15 \times 10^{-8} \pm 1.73 \times 10^{-9}$	$1.89 \times 10^{-9} \pm 8.34 \times 10^{-10}$	4.62×10^{-3}

Table S2. Summary of solution resistance, double layer capacitance and chi-square values of equivalent circuit fitting for EIS.

	α_{CPE} in $C_{DL,Pt}$	α_{CPE} in $C_{DL,5A1N}$	α_{CPE} in $C_{DL,Xero}$
Pt (n= 19)	$8.83 \times 10^{-1} \pm 5.36 \times 10^{-3}$	-	-
5A1N (n= 8)	$8.21 \times 10^{-1} \pm 3.12 \times 10^{-2}$	$7.39 \times 10^{-1} \pm 2.70 \times 10^{-2}$	-
Xerogel (n= 8)	$8.90 \times 10^{-1} \pm 3.58 \times 10^{-2}$	$8.89 \times 10^{-1} \pm 1.33 \times 10^{-2}$	$9.69 \times 10^{-1} \pm 2.28 \times 10^{-2}$

Table S3. Calculated constant phase element (CPE) using equivalent circuit models presented in Figure S5.

REFERENCE AND NOTES

1. P. Picón-Pagès, J. Garcia-Buendia, F. J. Munoz, Functions and dysfunctions of nitric oxide in brain. *Biochim. Biophys. Acta Mol. Basis Dis.* **1865**, 1949–1967 (2019).

2. B. V. Zlokovic, Neurovascular pathways to neurodegeneration in Alzheimer's disease and other disorders. *Nat. Rev. Neurosci.* **12**, 723–738 (2011).

3. M. Ziche, L. Morbidelli, Nitric oxide and angiogenesis. *J. Neuro-Oncol.* **50**, 139–148 (2000).

4. J. W. Coleman, Nitric oxide in immunity and inflammation. *Int. J. Immunopharmacol.* **1**, 1397–1406 (2001).

5. A. Phaniendra, D. B. Jestadi, L. Periyasamy, Free radicals: Properties, sources, targets, and their implication in various diseases. *Indian J. Clin. Biochem.* **30**, 11–26 (2015).

6. K. Chen, R. N. Pittman, A. S. Popel, Nitric oxide in the vasculature: Where does it come from and where does it go? A quantitative perspective. *Antioxid. Redox Signal.* **10**, 1185–1198 (2008).

7. M. K. Meffert, B. A. Premack, H. Schulman, Nitric oxide stimulates Ca^{2+}-independent synaptic vesicle release. *Neuron* **12**, 1235–1244 (1994).

8. L. A. Ridnour, J. S. Isenberg, M. G. Espey, D. D. Thomas, D. D. Roberts, D. A. Wink, Nitric oxide regulates angiogenesis through a functional switch involving thrombospondin-1. *Proc. Natl. Acad. Sci. U.S.A.* **102**, 13147–13152 (2005).

9. F. C. Fang, Perspectives series: Host/pathogen interactions. Mechanisms of nitric oxide-related antimicrobial activity. *J. Clin. Invest.* **99**, 2818–2825 (1997).

10. M. Benhar, Emerging roles of protein s-nitrosylation in macrophages and cancer cells. *Curr. Med. Chem.* **23**, 2602–2617 (2016).

11. J. Gow, Y. Yang, M. Govindraj, C. Guo, Nitric oxide regulates macrophage fungicidal activity via *S*-nitrosylation of dectin-1. *Appl. Vitro Toxicol.* **6**, 90–98 (2020).

12. F. C. Fang, Antimicrobial reactive oxygen and nitrogen species: Concepts and controversies. *Nat. Rev. Microbiol.* **2**, 820–832 (2004).

13. M. B. Witte, A. Barbul, Role of nitric oxide in wound repair. *Am. J. Surg.* **183**, 406–412 (2002).

14. M. Rizk, M. B. Witte, A. Barbul, Nitric oxide and wound healing. *World J. Surg.* **28**, 301–306 (2004).

15. J.-D. Luo, A. F. Chen, Nitric oxide: A newly discovered function on wound healing. *Acta Pharmacol. Sin.* **26**, 259–264 (2005).

16. J. V. Boykin Jr., Wound nitric oxide bioactivity: A promising diagnostic indicator for diabetic foot ulcer management. *J. Wound Ostomy Continence Nurs.* **37**, 25–32 (2010).

17. F. Yang, X. Bai, X. Dai, Y. Li, The biological processes during wound healing. *Regen. Med.* **16**, 373–390 (2021).

18. J. S. Isenberg, L. A. Ridnour, M. G. Espey, D. A. Wink, D. A. Roberts, Nitric oxide in wound-healing. *Microsurgery* **25**, 442–451 (2005).

19. P. Martin, R. Nunan, Cellular and molecular mechanisms of repair in acute and chronic wound healing. *Br. J. Dermatol.* **173**, 370–378 (2015).

20. S. Werner, R. Grose, Regulation of wound healing by growth factors and cytokines. *Physiol. Rev.* **83**, 835–870 (2003).

21. N. Lohmann, L. Schirmer, P. Atallah, E. Wandel, R. A. Ferrer, C. Werner, J. C. Simon, S. Franz, U. Freudenberg, Glycosaminoglycan-based hydrogels capture inflammatory chemokines and rescue defective wound healing in mice. *Sci. Transl. Med.* **9**, eaai9044 (2017).

22. Y. Jiang, A. A. Trotsyuk, S. Niu, D. Henn, K. Chen, C.-C. Shih, M. R. Larson, A. M. Mermin-Bunnell, S. Mittal, J.-C. Lai, A. Saberi, E. Beard, S. Jing, D. Zhong, S. R. Steele, K. Sun, T. Jain, E. Zhao, C. R. Neimeth, W. G. Viana, J. Tang, D. Sivaraj, J. Padmanabhan, M.

Rodrigues, D. P. Perrault, A. Chattopadhyay, Z. N. Maan, M. C. Leeolou, C. A. Bonham, S. H. Kwon, H. C. Kussie, K. S. Fischer, G. Gurusankar, K. Liang, K. Zhang, R. Nag, M. P. Snyder, M. Januszyk, G. C. Gurtner, Z. Bao, Wireless, closed-loop, smart bandage with integrated sensors and stimulators for advanced wound care and accelerated healing. *Nat. Biotechnol.* **41**, 652–662 (2023).

23. J. Shi, S. Kim, P. Li, F. Dong, C. Yang, B. Nam, C. Han, E. Eig, L. L. Shi, S. Niu, J. Yue, B. Tian, Active biointegrated living electronics for managing inflammation. *Science* **384**, 1023–1030 (2024).

24. J. W. Song, H. Ryu, W. Bai, Z. Xie, A. Vázquez-Guardado, K. Nandoliya, R. Avila, G. Lee, Z. Song, J. Kim, M. K. Lee, Y. Liu, M. Kim, H. Wang, Y. Wu, H. J. Yoon, S. S. Kwak, J. Shin, K. Kwon, W. Lu, X. Chen, Y. Huang, G. A. Ameer, J. A. Rogers, Bioresorbable, wireless, and battery-free system for electrotherapy and impedance sensing at wound sites. *Sci. Adv.* **9**, eade4687 (2023).

25. A. Tamayol, M. Akbari, Y. Zilberman, M. Comotto, E. Lesha, L. Serex, S. Bagherifard, Y. Chen, G. Fu, S. K. Ameri, W. Ruan, E. L. Miller, M. R. Dokmeci, S. Sonkusale, A. Khademhosseini, Flexible pH-sensing hydrogel fibers for epidermal applications. *Adv. Healthc. Mater.* **5**, 711–719 (2016).

26. H. Ryu, J. W. Song, H. Luan, Y. Sim, S. S. Kwak, H. Jang, Y. J. Jo, H. J. Yoon, H. Jeong, J. Shin, D. Y. Park, K. Kwon, G. A. Ameer, J. A. Rogers, Materials and device designs for wireless monitoring of temperature and thermal transport properties of wound beds during healing. *Adv. Healthc. Mater.* **13**, e2302797 (2024).

27. R. Zamora, Y. Vodovotz, T. R. Billiar, Inducible nitric oxide synthase and inflammatory diseases. *Mol. Med.* **6**, 347–373 (2000).

28. C. F. Nathan, J. B. Hibbs Jr., Role of nitric oxide synthesis in macrophage antimicrobial activity. *Curr. Opin. Immunol.* **3**, 65–70 (1991).

29. M. J. Malone-Povolny, S. E. Maloney, M. H. Schoenfisch, Nitric oxide therapy for diabetic wound healing. *Adv. Healthc. Mater.* **8**, e1801210 (2019).

30. C. N. Hall, J. Garthwaite, What is the real physiological NO concentration in vivo? *Nitric oxide* **21**, 92–103 (2009).

31. M. D. Brown, M. H. Schoenfisch, Electrochemical nitric oxide sensors: Principles of design and characterization. *Chem. Rev.* **119**, 11551–11575 (2019).

32. M. W. Vaughn, L. Kuo, J. C. Liao, Effective diffusion distance of nitric oxide in the microcirculation. *Am. J. Physiol.* **274**, H1705–H1714 (1998).

33. J. Lancaster Jr., A tutorial on the diffusibility and reactivity of free nitric oxide. *Nitric Oxide* **1**, 18–30 (1997).

34. F. Bedioui, S. Griveau, Electrochemical detection of nitric oxide: Assessement of twenty years of strategies. *Electroanalysis* **25**, 587–600 (2013).

35. C. D. Flynn, D. Chang, A. Mahmud, H. Yousefi, J. Das, K. T. Riordan, E. H. Sargent, S. O. Kelley, Biomolecular sensors for advanced physiological monitoring. *Nat. Rev. Bioeng.* **1**, 560–575 (2023).

36. S. Hassan, C. C. Schreib, X. Zhao, G. Duret, D. S. Roman, V. Nair, T. Cohen-Karni, O. Veiseh, J. T. Robinson, Real-time in vivo sensing of nitric oxide using photonic microring resonators. *ACS Sens.* **7**, 2253–2261 (2022).

37. J. Meier, J. Stapleton, E. Hofferber, A. Haworth, S. Kachman, N. M. Iverson, Quantification of nitric oxide concentration using single-walled carbon nanotube sensors. *Nanomaterials* **11**, 243 (2021).

38. B. J. Privett, J. H. Shin, M. H. Schoenfisch, Electrochemical nitric oxide sensors for physiological measurements. *Chem. Soc. Rev.* **39**, 1925–1935 (2010).

39. Y. Wang, S. Hu, Nitric oxide sensor based on poly (p-phenylenevinylene) derivative modified electrode and its application in rat heart. *Bioelectrochemistry* **74**, 301–305 (2009).

40. R. Li, H. Qi, Y. Ma, Y. Deng, S. Liu, Y. Jie, J. Jing, J. He, X. Zhang, L. Wheatley, C. Huang, X. Sheng, M. Zhang, L. Yin, A flexible and physically transient electrochemical sensor for real-time wireless nitric oxide monitoring. *Nat. Commun.* **11**, 3207 (2020).

41. Y. Deng, H. Qi, Y. Ma, S. Liu, M. Zhao, Z. Guo, Y. Jie, R. Zheng, J. Jing, K. Chen, H. Ding, G. Lv, K. Zhang, R. Li, H. Cheng, L. Zhao, X. Sheng, M. Zhang, L. Yin, A flexible and highly sensitive organic electrochemical transistor-based biosensor for continuous and wireless nitric oxide detection. *Proc. Natl. Acad. Sci. U.S.A.* **119**, e2208060119 (2022).

42. M. D. Brown, M. H. Schoenfisch, Selective and sensocompatible electrochemical nitric oxide sensor with a bilaminar design. *ACS Sens.* **4**, 1766–1773 (2019).

43. M. D. Brown, M. H. Schoenfisch, Nitric oxide permselectivity in electropolymerized films for sensing applications. *ACS Sens.* **1**, 1453–1461 (2016).

44. E. M. Hetrick, M. H. Schoenfisch, Analytical chemistry of nitric oxide. *Annu. Rev. Anal. Chem.* **2**, 409–433 (2009).

45. T. Xu, N. Scafa, L. P. Xu, L. Su, C. Li, S. Zhou, Y. Liu, X. Zhang, Electrochemical sensors for nitric oxide detection in biological applications. *Electroanalysis* **26**, 449–468 (2014).

46. T. Malinski, S. Mesaros, P. Tomboulian, "Nitric oxide measurement using electrochemical methods" in *Methods in Enzymology.* (Elsevier, 1996), vol. 268, pp. 58–69.

47. X. Zhang, Real time and in vivo monitoring of nitric oxide by electrochemical sensors—From dream to reality. *Front. Biosci.* **9**, 17 (2004).

48. S. S. Park, J. Kim, Y. Lee, Improved electrochemical microsensor for the real-time simultaneous analysis of endogenous nitric oxide and carbon monoxide generation. *Anal. Chem.* **84**, 1792–1796 (2012).

49. T. Malinski, Z. Taha, Nitric oxide release from a single cell measured in situ by a porphyrinic-based microsensor. *Nature* **358**, 676–678 (1992).

50. J. S. Reichner, A. J. Meszaros, C. A. Louis, W. L. Henry Jr., B. Mastrofrancesco, B.-A. Martin, J. E. Albina, Molecular and metabolic evidence for the restricted expression of inducible nitric oxide synthase in healing wounds. *Am. J. Physiol.* **154**, 1097–1104 (1999).

51. S. Frank, J. Pfeilschifter, M. Madlener, S. Werner, Induction of inducible nitric oxide synthase and its corresponding tetrahydrobiopterin-cofactor-synthesizing enzyme GTP-cyclohydrolase I during cutaneous wound repair. *J. Investig. Dermatol.* **111**, 1058–1064 (1998).

52. K. Moore, R. McCallion, R. J. Searle, M. C. Stacey, K. G. Harding, Prediction and monitoring the therapeutic response of chronic dermal wounds. *Int. Wound J.* **3**, 89–98 (2006).

53. S. Li, A. H. Mohamedi, J. Senkowsky, A. Nair, L. Tang, Imaging in chronic wound diagnostics. *Adv. Wound Care* **9**, 245–263 (2020).

54. T. Malinski, Z. Taha, S. Grunfeld, S. Patton, M. Kapturczak, P. Tomboulian, Diffusion of nitric oxide in the aorta wall monitored in situ by porphyrinic microsensors. *Biochem. Biophys. Res. Commun.* **193**, 1076–1082 (1993).

55. J. H. Shin, B. J. Privett, J. M. Kita, R. M. Wightman, M. H. Schoenfisch, Fluorinated xerogel-derived microelectrodes for amperometric nitric oxide sensing. *Anal. Chem.* **80**, 6850–6859 (2008).

56. E. P. Cintra, S. I. Córdoba de Torresi, N. Errien, G. Louarn, Determination of the formation of ladder structure in poly (5-amino-1-naphthol) by resonant Raman and XPS characterization. *Macromolecules* **36**, 2079–2084 (2003).

57. M. Mostefai, M. C. Pham, J. P. Marsault, J. Aubard, P. C. Lacaze, Study of the redox process of poly (5-amino-1-naphthol) thin film by in situ Raman spectroscopy. *J. Electrochem. Soc.* **143**, 2116–2119 (1996).

58. E. P. Cintra, S. I. C. de Torresi, Resonant Raman spectroscopy as a tool for determining the formation of a ladder structure in electropolymerized poly (5-amino-1-naphthol). *J. Electroanal. Chem.* **518**, 33–40 (2002).

59. R. Hemley, H. Mao, P. Bell, B. Mysen, Raman spectroscopy of si o 2 glass at high pressure. *Phys. Rev. Lett.* **57**, 747–750 (1986).

60. W. R. Thompson, J. E. Pemberton, Surface Raman scattering of self-assembled monolayers of (3-mercaptopropyl) trimethoxysilane on silver: Orientational effects of hydrolysis and condensation reactions. *Chem. Mater.* **5**, 241–244 (1993).

61. S. W. Kang, O. K. Kim, B. Seo, S. H. Lee, F. S. Quan, J. H. Shin, G.-J. Lee, H.-K. Park, Simultaneous, real-time measurement of nitric oxide and oxygen dynamics during cardiac ischemia–reperfusion of the rat utilizing sol–gel-derived electrochemical microsensors. *Anal. Chim. Acta* **802**, 74–81 (2013).

62. C. Boehler, S. Carli, L. Fadiga, T. Stieglitz, M. Asplund, Tutorial: Guidelines for standardized performance tests for electrodes intended for neural interfaces and bioelectronics. *Nat. Protoc.* **15**, 3557–3578 (2020).

63. R. Garg, G. Balakrishnan, R. B. Rashid, S. A. Gershanok, D. S. Roman, Y. Wang, P. C. Kouassi, J. Rivnay, T. Cohen-Karni, Graphene and Poly (3, 4-ethylenedioxythiophene)–polystyrene sulfonate hybrid nanostructures for input/output bioelectronics. *ACS Appl. Nano Mater.* **6**, 8495–8505 (2023).

64. A. J. Bard, L. R. Faulkner, H. S. White, *Electrochemical Methods: Fundamentals and Applications* (John Wiley & Sons, 2022).

65. T. Alencherry, A. Naveen, S. Ghosh, J. Daniel, R. Venkataraghavan, Effect of increasing electrical conductivity and hydrophilicity on the electrosorption capacity of activated carbon electrodes for capacitive deionization. *Desalination* **415**, 14–19 (2017).

66. A. Shaver, S. D. Curtis, N. Arroyo-Curras, Alkanethiol monolayer end groups affect the long-term operational stability and signaling of electrochemical, aptamer-based sensors in biological fluids. *ACS Appl. Mater. Interfaces* **12**, 11214–11223 (2020).

67. J. Veselinovic, S. AlMashtoub, S. Nagella, E. Seker, Interplay of effective surface area, mass transport, and electrochemical features in nanoporous nucleic acid sensors. *Anal. Chem.* **92**, 10751–10758 (2020).

68. A. U. Svensby, E. Nygren, A. Gefen, B. Cullen, Å. M. Ronkvist, A. Gergely, M. D. Craig, The importance of the simulated wound fluid composition and properties in the determination of the fluid handling performance of wound dressings. *Int. Wound J.* **21**, e14861 (2024).

69. T. Sridara, J. Upan, G. Saianand, A. Tuantranont, C. Karuwan, J. Jakmunee, Non-enzymatic amperometric glucose sensor based on carbon nanodots and copper oxide nanocomposites electrode. *Sensors* **20**, 808 (2020).

70. J. Wang, *Analytical Electrochemistry* (Wiley, ed. 3, 2006).

71. C. Bogdan, Nitric oxide and the immune response. *Nat. Immunol.* **2**, 907–916 (2001).

72. M. Wu, Z. Lu, K. Wu, C. Nam, L. Zhang, J. Guo, Recent advances in the development of nitric oxide-releasing biomaterials and their application potentials in chronic wound healing. *J. Mater. Chem. B* **9**, 7063–7075 (2021).

73. D. T. Efron, D. Most, A. Barbul, Role of nitric oxide in wound healing. *Curr. Opin. Clin. Nutr. Metab. Care* **3**, 197–204 (2000).

74. N. X. Landén, D. Li, M. Ståhle, Transition from inflammation to proliferation: A critical step during wound healing. *Cell Mol. Life Sci.* **73**, 3861–3885 (2016).

75. J. Dou, R. Yang, X. Jin, P. Li, X. Han, L. Wang, B. Chi, J. Shen, J. Yuan, Nitric oxide-releasing polyurethane/S-nitrosated keratin mats for accelerating wound healing. *Regenerative. Biomaterials* **9**, rbac006 (2022).

76. K. H. Hu, N. F. Kuhn, T. Courau, J. Tsui, B. Samad, P. Ha, J. R. Kratz, A. J. Combes, M. F. Krummel, Transcriptional space-time mapping identifies concerted immune and stromal cell patterns and gene programs in wound healing and cancer. *Cell Stem Cell* **30**, 885–903.e10 (2023).

77. D. S. Foster, M. Januszyk, K. E. Yost, M. S. Chinta, G. S. Gulati, A. T. Nguyen, A. R. Burcham, A. Salhotra, R. C. Ransom, D. Henn, K. Chen, S. Mascharak, K. Tolentino, A. L. Titan, R. E. Jones, O. da Silva, W. T. Leavitt, C. D. Marshall, H. E. des Jardins-Park, M. S. Hu, D. C. Wan, G. Wernig, D. Wagh, J. Coller, J. A. Norton, G. C. Gurtner, A. M. Newman, H. Y. Chang, M. T. Longaker, Integrated spatial multiomics reveals fibroblast fate during tissue repair. *Proc. Natl. Acad. Sci. U.S.A.* **118**, e2110025118 (2021).

78. M. R. Schäffer, U. Tantry, S. S. Gross, H. L. Wasserkrug, A. Barbul, Nitric oxide regulates wound healing. *J. Surg. Res.* **63**, 237–240 (1996).

79. D. M. Mosser, J. P. Edwards, Exploring the full spectrum of macrophage activation. *Nat. Rev. Immunol.* **8**, 958–969 (2008).

80. D.-H. Kim, J. Viventi, J. J. Amsden, J. Xiao, L. Vigeland, Y.-S. Kim, J. A. Blanco, B. Panilaitis, E. S. Frechette, D. Contreras, D. L. Kaplan, F. G. Omenetto, Y. Huang, K. C. Hwang, M. R. Zakin, B. Litt, J. A. Rogers, Dissolvable films of silk fibroin for ultrathin conformal bio-integrated electronics. *Nat. Mater.* **9**, 511–517 (2010).

81. W. D. Callister Jr, D. G. Rethwisch, *Materials Science and Engineering: An Introduction* (John wiley & sons, 2020).

82. T. Xu, J. H. Yoo, S. Babu, S. Roy, J.-B. Lee, H. Lu, Characterization of the mechanical behavior of SU-8 at microscale by viscoelastic analysis. *J. Micromech. Microeng.* **26**, 105001 (2016).

83. K. Wouters, R. Puers, Determining the Young's modulus and creep effects in three different photo definable epoxies for MEMS applications. *Sens. Actuators A: Phys.* **156**, 196–200 (2009).

84. B. Gorman, R. A. Orozco-Teran, J. A. Roepsch, H. Dong, R. F. Reidy, D. Mueller, High strength, low dielectric constant fluorinated silica xerogel films. *Appl. Phys. Lett.* **79**, 4010–4012 (2001).

85. R. M. Santos, M. S. Rodrigues, J. Laranjinha, R. M. Barbosa, Biomimetic sensor based on hemin/carbon nanotubes/chitosan modified microelectrode for nitric oxide measurement in the brain. *Biosens. Bioelectron.* **44**, 152–159 (2013).

86. S. Jiang, R. Cheng, X. Wang, T. Xue, Y. Liu, A. Nel, Y. Huang, X. Duan, Real-time electrical detection of nitric oxide in biological systems with sub-nanomolar sensitivity. *Nat. Commun.* **4**, 2225 (2013).

87. Y. Ha, J. Sim, Y. Lee, M. Suh, Insertable fast-response amperometric NO/CO dual microsensor: Study of neurovascular coupling during acutely induced seizures of rat brain cortex. *Anal. Chem.* **88**, 2563–2569 (2016).

88. J. Moon, Y. Ha, M. Kim, J. Sim, Y. Lee, M. Suh, Dual electrochemical microsensor for real-time simultaneous monitoring of nitric oxide and potassium ion changes in a rat brain during spontaneous neocortical epileptic seizure. *Anal. Chem.* **88**, 8942–8948 (2016).

89. O. Simoska, J. Duay, K. J. Stevenson, Electrochemical detection of multianalyte biomarkers in wound healing efficacy. *ACS Sens.* **5**, 3547–3557 (2020).
